# From exhaustion to functional cure: frontiers in reversing HBV-specific T-cell immunity

**DOI:** 10.3389/fimmu.2026.1877059

**Published:** 2026-07-06

**Authors:** Lifen Liang, Xiangping Xie, Shuangyan He, Liang Zhou, Yehong Yue

**Affiliations:** 1 Department of Infectious Disease, Shaoyang Central Hospital, Shaoyang, China; 2 Department of General Medicine, the Second Affiliated Hospital of Wannan Medical College, Wuhu, China

**Keywords:** chronic hepatitis B, combination therapy, epigenetic reprogramming, functional cure, immune checkpoint blockade, metabolic rewiring, T-cell exhaustion, TOX

## Abstract

Chronic hepatitis B (CHB) remains a leading cause of liver-related morbidity and mortality, affecting approximately 254 million people worldwide and accounting for more than 800,000 deaths annually. Although nucleos(t)ide analogues (NAs) durably suppress viral replication, the annual rate of HBsAg loss is only ~1%, leaving functional cure as the foremost unmet clinical need. Exhaustion of HBV-specific CD8^+^ T cells is now widely accepted as the central immunological barrier to viral clearance. Single-cell sequencing, spatial transcriptomics and epigenome profiling have collectively redefined this state: T-cell dysfunction in CHB is not a uniform “exhaustion phenotype” but a profoundly heterogeneous condition reinforced at transcriptional, epigenetic, metabolic and microenvironmental levels. In this review, we dissect the molecular signatures and inter- and intra-patient heterogeneity of HBV-specific T-cell exhaustion, with emphasis on the exhaustion programmes orchestrated by TOX, NFAT and NR4A factors, the epigenetic “lock-in” imposed by DNA methylation and histone modifications, and the bioenergetic insufficiency that arises from mitochondrial dysfunction. We then critically evaluate current reversal strategies, including immune checkpoint blockade (ICB), epigenetic modulators, engineered cytokines, microenvironment-directed agents (notably the ECM1–latent TGF-β axis), metabolic rewiring, and CAR-T/TCR-T cell therapy. Particular attention is given to the 2024 landmark studies that reshaped the conceptual landscape of the field — 4-1BB/OX40 co-stimulation, the hepatic immune rheostat, and attenuated effector T cells. We argue that no single intervention will suffice; only combinatorial regimens that simultaneously unlock epigenetic constraints, restore metabolic fitness, relieve checkpoint suppression and remodel the hepatic microenvironment can realistically advance HBV functional cure. This review is intended as a mechanism-grounded yet translationally oriented reference for both basic immunologists and hepatologists.

## 1 Introduction

Chronic hepatitis B (CHB) remains one of the heaviest public-health burdens worldwide. According to the 2024 Global Hepatitis Report from the World Health Organization, an estimated 254 million people are chronically infected with hepatitis B virus (HBV), with around 1.2 million new infections each year and more than 800,000 annual deaths attributable to HBV-related cirrhosis, hepatic failure and hepatocellular carcinoma (HCC) (1). Despite widespread vaccination, perinatal mother-to-child transmission, iatrogenic exposure and blood-borne transmission have not been eradicated, particularly in low- and middle-income regions (2, 3). China alone harbours an estimated 80 million chronically infected individuals (3). Current antiviral therapy is dominated by NAs (entecavir, tenofovir alafenamide) and pegylated interferon-α (PegIFN-α). NAs durably suppress HBV DNA in more than 95% of treated patients, but the annual rate of HBsAg loss is only 1–2%; PegIFN-α achieves somewhat higher response rates, although these vary widely by ethnicity, viral genotype, baseline HBsAg level and intrahepatic inflammation (4–6).

“Functional cure” has crystallised as the central therapeutic goal in CHB. Major international guidelines (EASL 2025, AASLD 2018) define it as sustained off-treatment HBsAg loss (with or without anti-HBs seroconversion), undetectable HBV DNA, normalisation of hepatic inflammation and absence of progression to cirrhosis or HCC (4, 5, 7, 8). Reaching this endpoint requires more than viral suppression: the host immune response to HBV must be fundamentally remodelled (9, 10). A new wave of mechanistically distinct agents — small interfering RNAs (siRNAs), antisense oligonucleotides (ASOs), capsid assembly modulators (CAMs), HBsAg secretion inhibitors (NAPs) and entry inhibitors such as bulevirtide — can drastically lower circulating viral antigens and thereby create an “immunological window” (11–13). However, these agents do not, on their own, reverse exhaustion in HBV-specific T cells. How best to integrate immune-directed therapy with antigen-reduction platforms therefore represents one of the most challenging and consequential questions in the field.

HBV-specific T cells are decisive for viral clearance (14–18). Building on the seminal work of Chisari and Guidotti, CD8+ T cells have been shown both to eliminate infected hepatocytes through cytolytic killing and to restrict cccDNA transcription and pgRNA translation through non-cytolytic mechanisms involving IFN-γ and TNF-α (19–22). In acute, self-limiting infection, the HBV-specific CD8+ T-cell response is polyclonal, multi-epitope and vigorous, with robust IFN-γ and TNF-α secretion alongside granzyme B/perforin-mediated cytotoxicity (23–25). By contrast, in chronic infection HBV-specific T cells are scarce, phenotypically dysfunctional, and persistently express multiple inhibitory receptors (PD-1, CTLA-4, TIM-3, LAG-3, TIGIT and others), with progressive loss of effector functions — the hallmarks of exhaustion (8, 26, 27).

The term “T-cell exhaustion” was first coined by Zajac and Gallimore in chronic LCMV infection (28, 29) and was later formalised by Wherry and colleagues as a distinct differentiation trajectory, separable from effector and memory states by transcriptional, epigenetic and metabolic features (30–33). The concept rapidly extended to chronic HCV, HIV and tumour immunology (34–37). In CHB, the seminal observation by Boni and colleagues that PD-1 blockade could partially restore HBV-specific CD8+ T-cell function (38, 39) catalysed two decades of mechanistic dissection — from single inhibitory receptors to the transcription factors TOX, NFAT and NR4A, and on to epigenetic reprogramming, metabolic insufficiency and the unique immunoregulatory landscape of the liver (27, 40–43).

Three landmark studies published in Cell, Nature and Nature Immunology in 2024 have profoundly reshaped this view (44–46). Andreata et al., using a functional screen of co-signalling receptors, showed that CD8+ T cells primed within the liver enter dysfunction immediately upon activation and that this state diverges substantially from the canonical LCMV exhaustion paradigm: blockade of inhibitory receptors such as PD-1 yields only modest benefit, whereas agonism of 4-1BB or OX40 reprogrammes these cells into bona fide antiviral effectors (44). Heim et al. defined an “attenuated effector” subset that retains partial cytolytic capacity but is restrained by TGF-β signalling — pinpointing TGF-β antagonism as a candidate target (45). Bosch et al. uncovered an entirely separate axis: liver sinusoidal endothelial cells (LSECs) drive enhanced CREM activity in CXCR6+ CD8+ T cells, rendering them unresponsive to TCR stimulation through a “hepatic immune rheostat” distinct from classical exhaustion (46). Together, these studies argue that T-cell dysfunction in HBV is not simply “canonical exhaustion” but an organ-tailored composite of liver-specific cues, intrinsic cellular programmes, epigenetic memory and microenvironmental signals (41, 47). Three further landmark contributions refine the exhaustion framework still further. Belk et al. used genome-wide CRISPR screens to map the regulators of progenitor-to-terminal exhaustion transitions and identify chromatin-remodelling complexes as gatekeepers of the fixed Ttex state (48). McManus et al. characterised a novel TOX-instructed enhancer programme that imprints inhibitory-receptor co-expression early in chronic stimulation and is partially reversible by combined epigenetic plus checkpoint intervention (49). Ghilas et al. defined the role of TCF1+ progenitor heterogeneity in conditioning the magnitude and durability of ICB responses, with direct implications for predicting reversibility in CHB (50). We have integrated these studies into Sections 2.2, 3.2 and 4.1 where the mechanistic continuity with HBV-specific T-cell biology is most direct. Building on this evidence, this review aims to provide both basic and translational investigators with a mechanistically grounded yet strategically oriented reference.

### 1.1 Literature search strategy

Because this review supports specific therapeutic recommendations, we have set out our literature search strategy explicitly. PubMed/MEDLINE, Web of Science and Embase were searched from inception to October 2025, with targeted updates of ClinicalTrials.gov and the EASL/AASLD congress abstract archives up to the same date. The principal search terms were combined as follows: (“hepatitis B” OR “HBV” OR “chronic hepatitis B”) AND (“T-cell exhaustion” OR “T cell dysfunction” OR “PD-1” OR “TOX” OR “TCF1” OR “Tpex” OR “Ttex” OR “immune checkpoint” OR “epigenetic” OR “DNMT” OR “HDAC” OR “TGF-β” OR “ECM1” OR “CAR-T” OR “TCR-T” OR “functional cure”). For the three 2024 landmark studies and very recent advances we additionally searched the journals Nature, Cell, Nature Immunology, Immunity, Journal of Hepatology, Hepatology and Gut by hand. Records were screened first by title and abstract, then by full text. We prioritised original mechanistic studies in human HBV and validated animal models (LCMV, AAV-HBV, humanised mice) and large randomised or controlled clinical trials over case series, conference abstracts and narrative reviews; abstracts and pre-prints were used only where no peer-reviewed publication was available, and are flagged as such in the text. Older mechanistic references were retained when they remain the primary source for a concept (for example, the Wherry, Zajac and Gallimore exhaustion definitions). Where preclinical and clinical evidence diverge, we have stated this explicitly so that recommendations are not over-extrapolated from animal data.

## 2 Features and assessment of T-cell exhaustion in HBV infection

### 2.1 Cardinal phenotypes of HBV-specific T-cell exhaustion

The classical definition of exhausted T cells originates from chronic LCMV infection and has since been refined in chronic HIV, HCV and tumour settings. Wherry and colleagues established the cardinal features: persistent high-level expression of inhibitory receptors, hierarchical loss of effector functions (IL-2 first, followed by TNF-α, and finally IFN-γ together with granzymes and perforin), reduced proliferative capacity, and heightened susceptibility to apoptosis (30, 32, 40, 51). Each of these features has been reproducibly documented in HBV (26, 27, 39, 52).

At the level of inhibitory receptors, HBV-specific CD8+ T cells co-express PD-1, CTLA-4, TIM-3, LAG-3, TIGIT, CD160 and 2B4 (39, 53–60). Early work by the Bertoletti and Maini groups identified PD-1 as the dominant exhaustion receptor in HBV-specific CD8+ T cells, with expression scaling alongside viral load, ALT and functional impairment (54, 55). Co-expression of TIM-3 and LAG-3 amplifies dysfunction: Nebbia et al. and Ye et al. demonstrated that PD-1^+^TIM-3^+^ or PD-1^+^LAG-3^+^ CD8+ T cells exhibit deeper IFN-γ deficits and greater proliferative impairment, and that combined blockade is synergistic (56, 57). TIGIT has more recently emerged as a clinically important inhibitor: Cai et al. showed that TIGIT^+^ CD8+ T cells are enriched in CHB and that disrupting the TIGIT–PVR axis partially rescues their function (58).

Effector loss in HBV-specific CD8+ T cells follows a graded, hierarchical pattern (27, 30). Park et al. used quantitative intracellular cytokine staining to show that IL-2 is the first cytokine extinguished, followed by TNF-α, and that IFN-γ disappears almost entirely in deeply exhausted cells (61). Granzyme B and perforin expression are markedly reduced, eroding direct cytolytic capacity. Intrahepatic exhaustion is consistently more severe than its peripheral counterpart, underlining the central role of the local microenvironment — rich in IL-10 and TGF-β, and dotted with PD-L1-high parenchymal and non-parenchymal cells — in maintaining the exhausted state (27, 62).

Reduced proliferative output and increased apoptotic susceptibility are further defining features (63–66). Lopes et al. showed that HBV-specific CD8+ T cells from CHB patients overexpress the pro-apoptotic protein Bim and rapidly undergo apoptosis upon antigen re-exposure, a key reason for their numerical scarcity (64). Peppa et al. extended this picture by demonstrating that NK cells actively delete HBV-specific T cells through TRAIL-mediated killing, adding a further layer of suppression (65). Mitochondrial dysfunction also imposes a substantial survival burden, as discussed below (67, 68).

High-dimensional cytometry and single-cell sequencing have uncovered substantial heterogeneity within HBV-specific CD8+ T cells. Bengsch and colleagues, using epigenome-guided mass cytometry (EpiCyTOF), defined disease-specific phenotypic combinations that grade exhaustion severity (69). Hoogeveen et al. found that the depth of exhaustion is epitope-dependent: core-specific CD8+ T cells retain comparatively better function, whereas polymerase-specific cells are the most profoundly exhausted (70). These observations make it clear that a single, monolithic “exhaustion” label fails to capture the complexity of the HBV-specific T-cell compartment.

### 2.2 Heterogeneity of exhaustion: Tpex and Ttex subsets in HBV

Exhausted T-cell populations are internally heterogeneous. In chronic LCMV and tumour models, at least two cardinal subsets have been delineated: a TCF1+ (encoded by TCF7) progenitor exhausted compartment (Tpex/T_pre-ex) and a terminally exhausted compartment (Ttex/T_term-ex) characterised by high PD-1, TIM-3 and CD101 but loss of TCF1 (71–76). Im et al. first showed that Tpex have superior self-renewal capacity and are the cellular substrate of the proliferative burst that follows PD-1 blockade (71). Beltra et al. further subdivided exhausted CD8+ T cells into four developmental stages: T_pex1 (CD69+Ly108+), T_pex2 (CD69−Ly108+), T_int (intermediate) and T_term (72).

**Figure 1:**
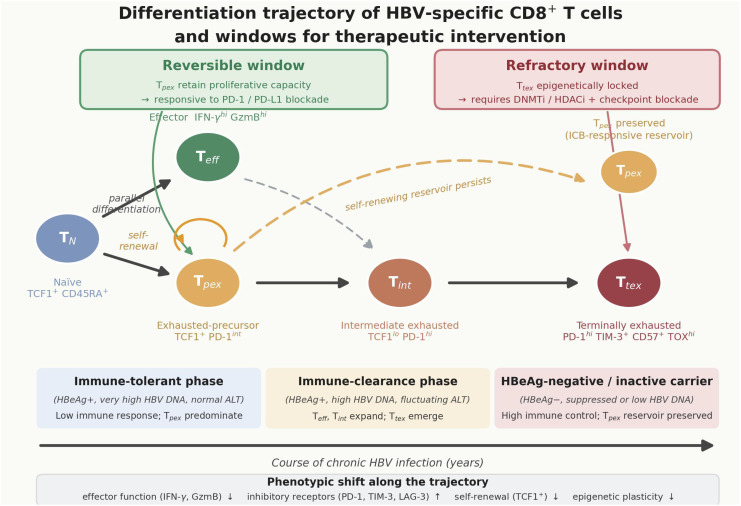
Differentiation trajectory of HBV-specific CD8^+^ T cells across the course of chronic infection and windows for therapeutic intervention. Following antigen encounter, naïve CD8^+^ T cells (T_N) differentiate in parallel along two arms: an effector arm (T_eff, IFN-γ^hi, GzmB^hi) and a precursor-exhausted arm (T_pex, TCF1^+^, PD-1^int, self-renewing). Consistent with chronic-LCMV studies, T_pex are generated early — in parallel with effectors, not as their descendants — and predominate in the immune-tolerant phase, where antigenaemia is highest and overt immune-mediated injury is minimal. As infection enters the immune-clearance phase, effector and intermediate exhausted (T_int) populations expand and terminally exhausted (T_tex, PD-1^hi TIM-3^+^ CD57^+^ TOX^hi) cells begin to emerge, accompanying the ALT flares and partial loss of HBV antigens (e.g. through precore/basal-core-promoter mutations). In the HBeAg-negative inactive carrier phase, viral load is suppressed and immune control is at its highest; the T_pex pool is preserved as the ICB-responsive reservoir. T_pex retain proliferative capacity and respond to PD-1/PD-L1 blockade (reversible window), whereas T_tex are epigenetically locked and largely refractory to checkpoint inhibition alone, requiring prior or concomitant epigenetic intervention (DNMTi, HDACi) (refractory window). Effector output (IFN-γ, granzyme B) and self-renewal (TCF1^+^) decline along the trajectory, whereas inhibitory-receptor co-expression and epigenetic rigidity increase.

A similar but more intricate Tpex/Ttex architecture exists in HBV. Wieland and colleagues demonstrated that TCF1+ memory-like CD8+ T cells persist in chronic HCV after DAA cure, indicating that this compartment underpins long-term immune surveillance (77). In HBV, Schuch et al. showed that core-specific CD8+ T cells retain TCF1 to a greater extent than polymerase-specific cells and that this preservation correlates with lower viral load (78). Heim et al. (2021) used single-cell analysis to chart the dynamic shift of Tpex/Ttex ratios across CHB phases — immune-tolerant, immune-active, HBeAg-negative and inactive carrier — showing that Tpex predominate in the immune-tolerant phase whereas Ttex expand markedly during the immune-active and HBeAg-negative phases (79) (**Figure 1**).

The 2024 studies further refined this map. Andreata et al. systematically dissected the heterogeneity of intrahepatically primed HBV-specific CD8+ T cells and identified a long-lived, self-renewing dysfunctional “stem-like” population (TSL), together with two distinct dysfunctional tissue-resident memory (TRM) subsets (44). Critically, these populations responded poorly to ICB but robustly to 4-1BB or OX40 agonism, indicating that HBV-induced dysfunction is governed by intrinsic regulatory circuits that diverge from canonical exhaustion (44). The “attenuated effector” subset described by Heim et al. retains partial cytotoxicity but is constrained by TGF-β (45), and the CXCR6+CREMhigh population identified by Bosch et al. is silenced by an LSEC-driven hepatic immune rheostat (46). Together, these findings argue that future reversal strategies must rest on precise subset stratification rather than on undifferentiated rescue of an “exhausted” pool (41, 47).

The T_pex/T_tex framework is partially supported in HBV but two caveats remain. First, the number of HBV-specific T cells available for single-cell capture is often <100 per sample, limiting subset resolution and reproducibility across cohorts. Second, whether the canonical TCF1+ T_pex compartment captures the entirety of the ICB-responsive reservoir in CHB — or whether liver-specific TCF1-independent reservoirs (e.g., the TSL population identified by Andreata et al.) coexist — has not been settled. Cross-validation across cohorts and consensus on subset-defining marker panels are required before T_pex/T_tex stratification can guide treatment decisions.

### 2.3 Clinically relevant functional T-cell subsets

Identifying T-cell subsets that track with viral control is a prerequisite for individualised immunotherapy. Rivino et al. showed that the frequency of PD-1^+^CD38^+^HLA-DR^+^ CD8+ T cells correlated positively with HBsAg decline and viral control after NA cessation (80). It should be emphasised, however, that CD38, HLA-DR and PD-1 are all activation markers, and that this phenotypic signature is most typically associated with effector CD8+ T cells in acute or recently re-activated infection rather than with terminally exhausted cells; in the absence of additional inhibitory receptors and the exhaustion-defining transcription factor TOX, these cells cannot be classified as exhausted. The fact that they correlate with HBsAg decline suggests that they are mediating productive immune control rather than awaiting therapeutic reversal, and accordingly we no longer interpret this subset as a target for exhaustion-reversing intervention but rather as a biomarker of an ongoing antiviral response.

Le Bert et al. extended this framework across CHB phases: in HBeAg-negative patients, HBV-specific CD8+ T cells retain features amenable to immunomodulation, whereas the immune-tolerant phase is marked by oligoclonal, less recoverable repertoires (81). Salimzadeh et al. simultaneously profiled HBV-specific B cells and reported PD-1^+^ B cells with analogous suppression, suggesting that PD-1 blockade may simultaneously rescue both adaptive arms (82).

Beyond the PD-1^+^CD38^+^HLA-DR^+^ subset, additional candidate biomarkers include downregulated CD127 (IL-7Rα), reflecting impaired memory transition, and CD57 upregulation, indicative of replicative senescence (83, 84). TIGIT^+^CD96^+^ CD8+ T cells likewise track with viral persistence and may provide a biomarker for TIGIT-directed therapy (58). These markers are gradually maturing into a coherent system, but their clinical utility awaits prospective, longitudinal validation.

## 3 Molecular mechanisms of HBV-specific T-cell dysfunction: exhaustion and distinct exhaustion-independent programmes

### 3.1 Persistent antigen stimulation and the immune microenvironment: drivers of exhaustion and distinct dysfunction-promoting cues

Persistent antigen stimulation is widely regarded as the principal driver of T-cell exhaustion (31, 32, 84). In HBV this driver is unusually intense: serum HBsAg levels can reach 10^4^–10^5^ IU/mL, and most circulating HBsAg resides on subviral particles (SVPs) that outnumber complete virions by 10³–10^4^-fold (19, 83, 85). The resulting “antigen excess” imposes sustained, high-amplitude signalling through the T-cell receptor (TCR), engaging NFAT-dependent transcriptional programmes that initiate exhaustion (86). Schuch and colleagues compared core-specific and polymerase-specific CD8+ T cells in chronically infected patients and reported that, between these two specificities, polymerase-specific cells show the most severe functional impairment (78). This comparison is restricted to core- versus polymerase-specific T cells; envelope- and X-specific populations were not assessed in sufficient numbers, so the finding is best framed as a contrast between core and polymerase specificities rather than as a claim that polymerase-specific cells are the most exhausted of the entire HBV-specific repertoire. Notably, polymerase-specific CD8+ T cells targeting the 455/173 epitopes are not terminally exhausted but predominantly display a memory-like phenotype in chronic infection. Taken together, these data indicate a dose–response relationship between antigen exposure and the depth of functional impairment across these two specificities, without implying that polymerase-specific cells are uniformly terminally exhausted.

The hepatic microenvironment further reinforces tolerance (62, 87, 88). As the principal recipient of gut-derived antigens, the liver has evolved multiple tolerogenic mechanisms: LSECs and Kupffer cells, the resident professional antigen-presenting cells, express low levels of co-stimulatory molecules but high PD-L1, biasing T-cell encounters towards tolerance rather than productive priming (62). In CHB, this baseline tolerance is co-opted by the virus. Maier et al. first showed that PD-L1 is markedly upregulated on both parenchymal and non-parenchymal cells in HBV-infected liver and directly contributes to CD8+ T-cell suppression (55). Building on this, Bosch et al. (2024) demonstrated that LSECs drive enhanced CREM activity in HBV-specific CXCR6+ CD8+ T cells, generating a state of dysfunction that is mechanistically distinct from canonical exhaustion (46).

Regulatory T cells (Tregs) play a dual role in HBV immunopathology (89–92). Stoop et al. first reported elevated frequencies of CD4+CD25+Foxp3+ Tregs in CHB peripheral blood and liver, with suppressive activity tracking with HBV-specific T-cell hypofunction (89). Xu et al. showed that circulating and intrahepatic Treg levels correlate inversely with antiviral response (90). Tregs suppress effector function through IL-10 and TGF-β secretion as well as through CTLA-4-dependent contact mechanisms, all of which reinforce exhaustion (91, 93).

Myeloid-derived suppressor cells (MDSCs) constitute another pivotal microenvironmental component (94, 95). Pallett et al. first demonstrated that HBeAg drives expansion of granulocytic MDSCs (G-MDSCs), which suppress HBV-specific T cells through arginase-1 (Arg1)- and iNOS-mediated amino-acid depletion and oxidative-stress products (94). Pal and colleagues subsequently showed that MDSC-mediated suppression persists despite NA therapy and continues to support Treg-driven inhibition (95).

Soluble cytokines complete the picture. IL-10 and TGF-β are the dominant immunosuppressive signals in CHB (45, 93). IL-10, secreted by Tregs, Kupffer cells and Bregs, downregulates MHC class II, blunts dendritic-cell maturation, and impairs T-cell effector function (96). TGF-β acts both directly on T cells and through latent activation by αv integrins on hepatocytes and the extracellular matrix (97, 98). The 2024 study by Heim and colleagues placed TGF-β squarely at the centre of HBV-specific CD8+ T-cell dysfunction, demonstrating that the “attenuated effector” population retains cytolytic capacity but is muzzled by TGF-β signalling — flagging TGF-β antagonism as a tractable therapeutic axis (45).

Exhaustion in CHB is initiated by a convergence of three forces (**Table 1**). *Key drivers* — overwhelming antigenaemia (HBsAg and SVPs at 10³–10^4^ molar excess) combined with the liver’s constitutive tolerogenic disposition (LSEC- and Kupffer-cell-imposed PD-L1, T_reg- and MDSC-derived IL-10 and TGF-β). *Downstream effects* — sustained partnerless NFAT signalling that initiates the canonical exhaustion programme, compounded by the liver-specific CREM-driven “immune rheostat” uncovered by Bosch et al. *Therapeutic implications* — these layered drivers cannot be neutralised by any single intervention, providing the rationale for combining antigen-load reduction (NAs, siRNA) and microenvironment-directed agents (TGF-β antagonists, T_reg/MDSC modulators) with cell-intrinsic interventions such as ICB and epigenetic therapy.

**Table 1:** Hierarchical framework of HBV-specific CD8+ T-cell exhaustion.

Layer	Key driver(s)	Downstream effect	Liver/HBV-specific feature	Therapeutic implication
Antigenic layer	HBsAg/SVP overload, HBeAg	Sustained NFAT signalling; partnerless NFAT	10³–10^4^ molar excess of SVPs over virions	NAs, siRNA, NAP — antigen reduction
Microenvironmental layer	LSEC, Kupffer cells, Treg, MDSC, IL-10, TGF-β	Tolerogenic priming; CREM activation	Hepatic immune rheostat (Bosch 2024)	TGF-β antagonism; ECM1a; integrin β1 blockade (ATN-161)
Transcriptional layer	TOX, NFAT, NR4A1/2/3, BLIMP1; T-bet/EOMES imbalance	Inhibitory-receptor induction; effector-gene silencing	TOX expression scales with viraemia and HBeAg status	TOX knockdown; NR4A targeting; TCF1-centred reprogramming
Epigenetic layer	DNMT3A, HDACs, EZH2	Stable chromatin “scar”: PDCD1/HAVCR2 demethylated, IFNG/GZMB silenced	Persists for >6 months after antigen withdrawal (HCV evidence)	DNMTi (DAC); HDACi; EZH2i; dCas9-TET1 epigenetic editing
Metabolic layer	PD-1 signalling; Arg1/IDO/TDO; mitochondrial-dynamics disturbance	Bioenergetic collapse (low OXPHOS, ATP); high ROS	Hepatic amino-acid scarcity intensifies the lesion	PGC-1α agonism; NAC/MitoTEMPO; L-arginine; rapamycin tuning
Co-stimulatory layer	Reduced 4-1BB/OX40 engagement; ICOS imbalance	“Attenuated effector” state — partial cytotoxicity, TGF-β-restrained	Andreata 2024/Heim 2024 paradigm	4-1BB or OX40 agonism; OX40 + anti-PD-L1 combination

It is important to distinguish, however, between those mechanisms that drive cell-intrinsic, TOX-mediated exhaustion of HBV-specific CD8+ T cells and those that suppress T-cell function through routes that are mechanistically separate from canonical exhaustion. Findings of Heim et al. (2024) and Bosch et al. (2024), for example, describe an attenuated effector phenotype constrained by TGF-β and a CREM-driven LSEC rheostat, respectively, both of which operate at least partly independently of the TOX-driven exhaustion programme. Likewise, T_reg-mediated suppression (Stoop et al.) and MDSC-mediated amino-acid depletion (Pallett et al.) act on otherwise competent T cells from outside and would dampen function even in cells that are not transcriptionally locked into exhaustion. In the present review we therefore use “dysfunction” as the overarching term for the loss of HBV-specific T-cell function in chronic infection and reserve “exhaustion” for the cell-intrinsic, TOX-defined programme; the two are clearly related but should not be treated as synonymous, and this distinction is now reflected throughout Sections 3 and 4.

### 3.2 Transcription-factor circuitry

**Figure 2:**
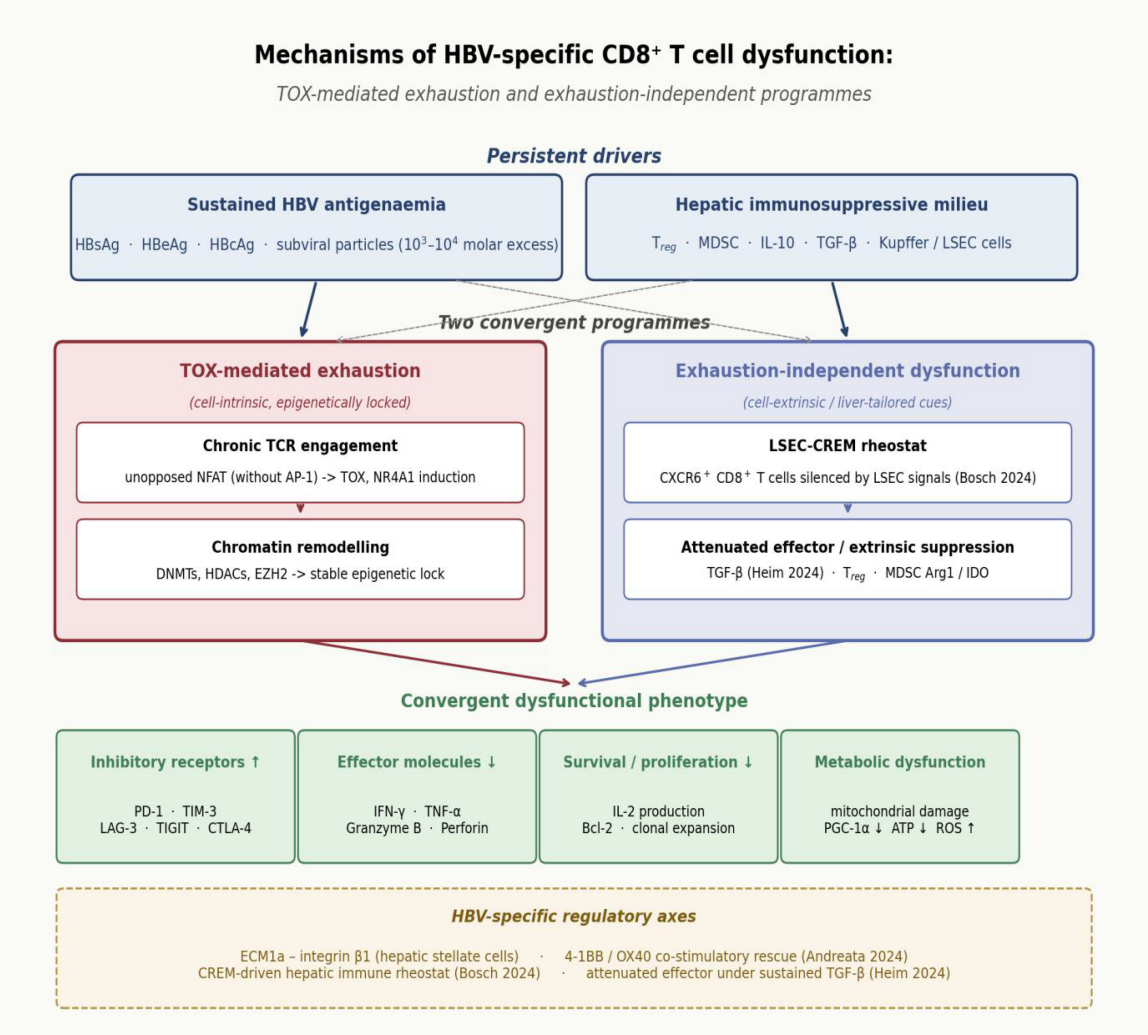
Central molecular circuitry of CD8^+^ T cell exhaustion in chronic HBV infection. Persistent HBV antigenaemia (HBsAg, HBeAg, HBcAg) together with the hepatic immunosuppressive milieu (T_reg cells, MDSCs, IL-10, TGF-β, Kupffer/LSEC cells) drives chronic TCR engagement and unopposed NFAT activity, inducing TOX as the master switch of the exhaustion programme. TOX recruits chromatin-modifying enzymes — DNMTs, HDACs and EZH2 — that remodel the epigenome and lock in lineage commitment. This TOX-driven programme is shown as the “exhaustion” arm, while T_reg/MDSC/IL-10/TGF-β/LSEC–CREM cues are grouped as a separate “exhaustion-independent dysfunction” arm that converges on the same phenotypic readouts. The resulting phenotype combines elevated inhibitory-receptor expression (PD-1, TIM-3, LAG-3, TIGIT, CTLA-4), blunted effector function (IFN-γ, TNF-α, granzyme B, perforin), defective survival and proliferation (IL-2, Bcl-2) and impaired mitochondrial bioenergetics (PGC-1α, ATP). Liver-tailored regulatory axes — the ECM1a–integrin β1 axis, the CREM-driven hepatic immune rheostat (Bosch et al., 2024), the attenuated effector programme imposed by sustained TGF-β (Heim et al., 2024) and the 4-1BB/OX40 co-stimulatory rescue (Andreata et al., 2024) — overlay this circuit and define HBV-specific therapeutic vulnerabilities.

Among the most consequential advances of the past decade is the elucidation of the transcription-factor network that programmes exhaustion (32, 42, 84). TOX (thymocyte selection-associated high mobility group box) is now firmly established as the master switch. The four landmark Nature studies of 2019 — by the Khan, Scott, Alfei and Yao groups — collectively defined a single, coherent model (42, 43, 99–102): persistent TCR-NFAT signalling induces TOX, which in turn remodels chromatin and recruits DNA methyltransferases (DNMTs) and histone deacetylases (HDACs) to lock T cells into the exhausted lineage (**Figure 2**).

Heim and colleagues were the first to extend the TOX axis to human HBV (79). They showed that TOX expression in HBV-specific CD8+ T cells scales with disease severity, peaks in HBeAg-positive, high-viraemia patients, and correlates strongly with PD-1 and inversely with TCF1. Crucially, knockdown of TOX partially restored IFN-γ and granzyme B production (79), establishing TOX as a tractable target in HBV.

NFAT and NR4A factors complete the core exhaustion circuit alongside TOX (86, 103–106). Martinez et al. (2015) showed that NFAT activation uncoupled from its AP-1 partners (c-Fos/c-Jun) is sufficient to drive an exhaustion programme rather than effector differentiation (86), explaining why sustained TCR signalling without appropriate co-stimulation paradoxically yields dysfunction. In this “partnerless” mode, NFAT directly transactivates PDCD1, HAVCR2, LAG3, TIGIT and TOX, while inducing NR4A1/2/3 to consolidate the programme (104, 105). Three concurrent studies in 2019 — by the Liu, Chen and Seo groups — confirmed that NR4A1, NR4A2 and NR4A3 cooperate with chromatin remodellers to entrench TOX-induced exhaustion (103–105).

TCF1 (TCF7) and BLIMP1 (PRDM1) embody the two opposing fate determinants in this circuit (31, 75, 107–110). TCF1 marks the Tpex compartment, conferring self-renewal and ICB-responsiveness, whereas BLIMP1 drives terminal differentiation accompanied by effector-molecule loss and proliferative collapse (31, 111, 112). Chen et al., using CRISPR screens, established TCF1 as the central node sustaining Tpex stemness through TOX repression (108). In HBV, the TCF1+ population, although small, is the cellular substrate of immune surveillance and ICB responsiveness (77, 79).

Beyond these core factors, T-bet and EOMES — classical drivers of effector and memory differentiation — display a characteristic imbalance in exhaustion: T-bet is reduced and EOMES is elevated, a configuration that marks terminal exhaustion (112, 113). Kao et al. showed that T-bet represses PD-1 expression and sustains CD8+ T-cell effector function; its loss accelerates exhaustion (111). The same imbalance contributes to HBV-specific CD8+ T-cell dysfunction (27).

It is important, however, to emphasise that the 2024 studies indicate that HBV-induced T-cell dysfunction does not strictly follow the classical exhaustion logic (41, 44, 47). Andreata et al. observed that intrahepatically primed HBV-specific CD8+ T cells upregulate multiple co-signalling receptors — PD-1, CTLA-4, LAG-3, OX40, 4-1BB and ICOS — from the earliest stages, yet single-receptor blockade (including PD-1) yields only modest functional rescue, whereas 4-1BB or OX40 agonism converts these cells into bona fide antiviral effectors (44). The implication is profound: HBV-specific T-cell dysfunction may be governed by liver-tailored transcriptional programmes that are not faithfully captured by the LCMV exhaustion paradigm, and a more refined map of organ-specific transcriptional control is now needed.

The exhaustion transcription-factor circuit can be reduced to a hierarchy: TOX as master switch → NFAT/NR4A as core reinforcers → TCF1/BLIMP1 as fate determinants → T-bet/EOMES as terminal-differentiation modulators. In CHB, TOX scales with disease severity and operates as a tractable molecular target. However, the 2024 evidence that HBV-specific dysfunction is not faithfully captured by the LCMV-derived TF model implies that organ-tailored transcription-factor circuits — particularly those engaging co-stimulatory receptors (4-1BB, OX40) and CREM — must be mapped before transcription-factor-directed therapy can reach the clinic.

### 3.3 Epigenetic reprogramming: the molecular shackles of exhaustion

**Figure 3:**
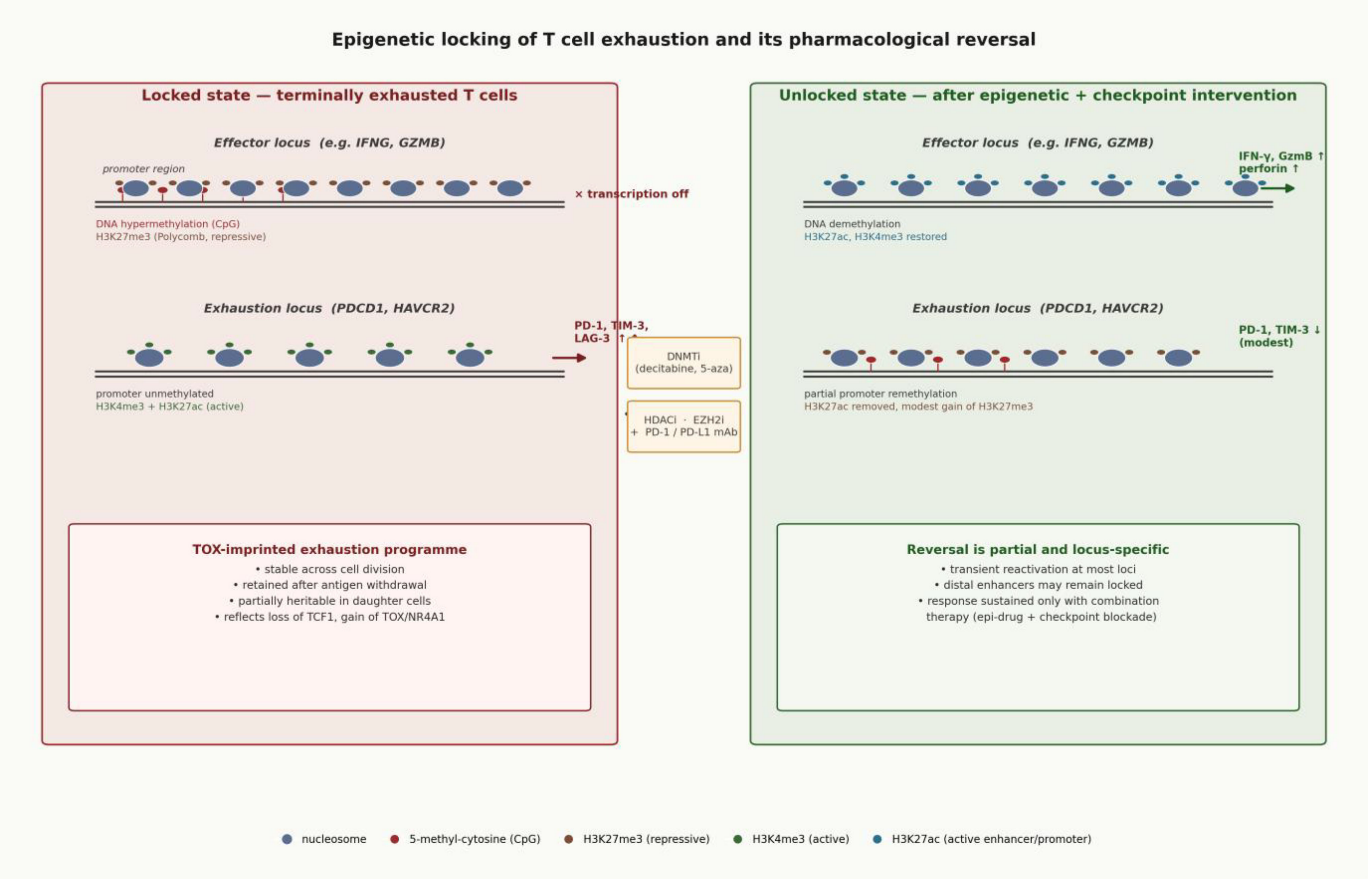
Epigenetic locking of T cell exhaustion and its pharmacological reversal. In terminally exhausted T cells (left), effector loci such as IFNG and GZMB carry promoter CpG hypermethylation and repressive H3K27me3 marks distributed across condensed nucleosomes, silencing transcription, whereas exhaustion-associated loci (PDCD1, HAVCR2) remain unmethylated and decorated with active H3K4me3 and H3K27ac, sustaining high inhibitory-receptor expression. This TOX-imprinted programme is heritable across cell divisions and is retained even after antigen withdrawal. Combined epigenetic plus checkpoint intervention (right) — DNMTi (decitabine, 5-azacytidine), HDAC inhibitors and EZH2 inhibitors together with PD-1/PD-L1 blockade — partially demethylates effector promoters, restores active histone marks and re-establishes effector transcription, while attenuating activity at exhaustion loci. Reversal is locus-specific and incomplete; some distal enhancers may remain stably locked, providing the rationale for combinatorial regimens to achieve durable functional rescue.

Epigenetic reprogramming is arguably the most distinctive — and the most durable — molecular hallmark of exhausted T cells (42, 113–115). The 2016 Science papers by Pauken, Sen and colleagues established the field-defining concept: exhausted T cells exhibit a characteristic chromatin-accessibility landscape and DNA-methylation profile that persist for months after antigen clearance (or PD-1 blockade), placing a ceiling on the extent of functional restoration (113, 114). Philip et al., using single-cell ATAC-seq, demonstrated that tumour-specific T cells transition from a plastic chromatin state in early dysfunction to a “fixed” terminal state that resists conventional immune intervention (116) (**Figure 3**).

DNA methylation features prominently in this lock-in (117–121). Youngblood and colleagues first showed that demethylation of the PDCD1 promoter underlies persistent PD-1 expression in chronic LCMV (118). Ahn et al. extended these findings to demonstrate that this demethylation is imprinted during the effector phase and persists long after antigen clearance, constituting an epigenetic memory of exhaustion (119). Conversely, the IFNG and GZMB loci accumulate methylation marks in their promoters and enhancers, transcriptionally silencing these effector genes (115, 119).

Histone modifications form a second layer of epigenetic locking (105, 115, 122). In exhausted CD8+ T cells, effector loci such as IFNG, TBX21 and GZMB lose H3K4me3 and gain H3K27me3, while inhibitory-receptor loci including PDCD1 and HAVCR2 show the opposite pattern, generating a stable “exhaustion epigenetic signature” (115). Henning and colleagues comprehensively reviewed the histone-modification landscape of exhaustion and identified EZH2-mediated H3K27me3 deposition as a critical mechanism for silencing effector genes (123).

The DAA-cure studies in HCV provided the most compelling clinical evidence for epigenetic locking. Yates et al., Hensel et al. and Tonnerre et al. independently showed that even six months after sustained virological response, HCV-specific CD8+ T cells retain prominent epigenetic “scars”: their DNA-methylation and chromatin-accessibility landscapes do not fully revert to a memory phenotype (124–126). Tonnerre and colleagues went further, defining these cells as “memory-like but trapped” — they acquire surface markers of memory but fail to complete the transcriptional and epigenetic transition to a fully functional memory state (126).

These findings carry sobering implications for HBV. If HCV cure — achieved through direct antiviral elimination — is insufficient to erase exhaustion-associated epigenetic memory, then HBsAg loss in CHB is unlikely, in itself, to fully restore HBV-specific T-cell function (42, 125). The corollary is that future cure strategies may need to combine antiviral therapy with epigenetic reprogramming agents capable of actively erasing exhaustion-associated marks. Ghoneim and colleagues provided proof-of-concept evidence in chronic LCMV: combination of the DNMT inhibitor decitabine with PD-L1 blockade was substantially more effective than either monotherapy (127).

Newer technologies such as EpiCyTOF (epigenome-guided mass cytometry) now allow exhaustion-specific epigenetic states to be inferred without sequencing (69). Bengsch and colleagues used this approach to identify disease-specific epigenetic signatures in CHB, providing a potentially translatable tool for clinical stratification (69).

Drivers — chronic TCR/TOX signalling recruits DNMTs, HDACs and EZH2 to inscribe a stable chromatin landscape. Downstream effects — PDCD1 and HAVCR2 are demethylated and decorated with active marks; IFNG, GZMB and TBX21 are hypermethylated and silenced by H3K27me3. Cross-disease evidence from HCV cure indicates that this “scar” persists for at least six months after antigen withdrawal. Therapeutic implications — antigen elimination alone is unlikely to restore function in CHB; functional cure will require active epigenetic erasure (DNMTi, HDACi, EZH2i, or locus-targeted dCas9-TET1) administered alongside or before checkpoint relief.

Three issues constrain the epigenetic-locking model in CHB. First, most evidence for epigenetic stability is derived from murine LCMV models or HCV post-DAA cohorts; whether human HBV-specific CD8+ T cells exhibit comparably “fixed” chromatin states *in vivo* has not been formally demonstrated, in part because of the extreme scarcity of intrahepatic HBV-specific T cells available for low-input epigenomic profiling. Second, the heterogeneity of CHB (HBeAg+ vs HBeAg−, high- vs low-viraemia, NA-treated vs untreated) is rarely controlled for in epigenomic datasets, so reported “exhaustion signatures” may amalgamate distinct subgroups. Third, the locus-specificity and reversibility of demethylation achievable with current DNMTi/HDACi dosing schedules in CHB are essentially undefined, raising the concern that genome-wide demethylation may reactivate proto-oncogenic loci in a population already at elevated HCC risk.

### 3.4 Metabolic rewiring: bioenergetic crisis and functional collapse

Metabolic rewiring is a third defining feature of exhausted T cells, intricately linked with both transcriptional and epigenetic programmes (67, 68, 128–130). Bengsch et al. analysed CD8+ T cells in chronic LCMV and showed that bioenergetic insufficiency precedes overt functional loss: oxidative phosphorylation (OXPHOS) is impaired, glycolytic capacity declines, and ATP production falls before effector deficits become detectable (67). This temporal precedence indicates that metabolic dysfunction is an early driver of exhaustion rather than a downstream consequence.

Mitochondrial dysfunction is the metabolic centrepiece of HBV-specific T-cell exhaustion (68). Fisicaro et al. were the first to document profound mitochondrial impairment in HBV-specific CD8+ T cells from CHB patients: depolarised membrane potential, elevated reactive oxygen species (ROS), and reduced expression of respiratory-chain components. Crucially, treatment with mitochondria-targeted antioxidants such as MitoTEMPO and N-acetylcysteine partially restored IFN-γ production and proliferative capacity, providing direct evidence that mitochondrial repair has therapeutic potential (68). Vardhana et al. extended these observations by showing that compromised OXPHOS limits ATP and NAD+ supply, thereby curtailing T-cell self-renewal under persistent antigen exposure (129).

PD-1 signalling itself contributes to metabolic remodelling, generating a feedback loop between exhaustion and energetic insufficiency (131). Patsoukis et al. demonstrated that PD-1 engagement suppresses glycolysis while promoting fatty-acid oxidation, thereby shaping the metabolic phenotype of exhausted cells (131). Bengsch and colleagues further showed that PD-1-driven mitochondrial dysfunction precedes overt exhaustion (67). The clinical implication is direct: PD-1 blockade alone is unlikely to fully restore metabolic fitness, and combined metabolic reprogramming may be required for optimal efficacy.

The unique metabolic landscape of the liver intensifies this energy crisis (62, 94). As the body’s metabolic hub, the liver is rich in arginase (Arg1), indoleamine 2,3-dioxygenase (IDO) and tryptophan 2,3-dioxygenase (TDO), all of which deplete amino acids essential for T-cell activation (94, 95). Pallett et al. showed that circulating G-MDSCs in CHB patients deplete extracellular arginine via Arg1 and that serum arginine is significantly reduced (94). Geiger and colleagues independently demonstrated that exogenous L-arginine improves T-cell metabolism and antitumour activity (132), pointing to amino-acid metabolism as a tractable axis for HBV-specific immune restoration.

Metabolic insufficiency and exhaustion also reinforce one another in a positive-feedback loop (133–136). Scharping et al. showed that hypoxia and nutrient deprivation in tumours suppress mitochondrial biogenesis through downregulation of PGC-1α, the master regulator of mitochondrial biogenesis, and that PGC-1α loss in turn reinforces the exhaustion programme — a vicious cycle (133). More recently, Yu and colleagues reported that chronic antigen stimulation profoundly disrupts mitochondrial dynamics, with abnormal fusion events generating dysfunctional mitochondrial clusters that further constrain T-cell recovery (135). Whether comparable mitochondrial-network disturbances perpetuate exhaustion in HBV is now a key open question (27, 68).

Mitochondrial bioenergetic insufficiency precedes overt effector loss and represents the earliest detectable molecular lesion of HBV-specific T-cell exhaustion. PD-1 signalling, hepatic amino-acid scarcity (Arg1, IDO, TDO) and disrupted mitochondrial dynamics together produce a self-reinforcing energy crisis. The therapeutic corollary is direct: restoring metabolic fitness (PGC-1α agonism, mitochondrial ROS scavenging, arginine repletion) should be considered a foundational layer of any combination regimen, on which checkpoint or epigenetic interventions can subsequently act.

Whether mitochondrial dysfunction is a primary driver or a secondary consequence of exhaustion in human CHB remains unresolved. The current evidence — depolarised membrane potential, elevated ROS, and partial rescue by MitoTEMPO or N-acetylcysteine — is largely cross-sectional and ex vivo, and longitudinal data linking mitochondrial recovery to *in vivo* HBsAg decline are absent. Furthermore, hepatic substrate scarcity (Arg1-driven arginine depletion, IDO/TDO-mediated tryptophan catabolism) overlaps with mechanisms operating in HCC and HCV, so the HBV-specific contribution of each axis has not been formally dissected.

### 3.5 HBV-specific liver-tailored regulatory axes: ECM1/TGF-β and emerging targets

The peculiar immunology of the liver imposes distinctive regulatory features on HBV-specific T cells that have no straightforward equivalent in LCMV or tumour models (41, 45, 46). Several liver-tailored axes — most notably the ECM1/latent-TGF-β activation axis and the hepatic immune rheostat — have come into sharp focus, and are reshaping the therapeutic agenda.

Extracellular matrix protein 1 (ECM1) deserves particular attention. Fan and colleagues, in a landmark Gastroenterology paper, established the role of ECM1 in hepatic homeostasis (97): produced predominantly by hepatocytes, ECM1 binds the αv-integrin recognition site of the latent TGF-β complex (LTC), thereby keeping TGF-β in its inactive form and protecting the liver from TGF-β-driven fibrosis and immunosuppression. Ecm1-knockout mice develop spontaneous severe hepatic fibrosis and die before two months of age, a phenotype rescued by AAV-mediated hepatocyte-specific ECM1 overexpression or soluble TGFβR2 (97). In human chronic liver disease and experimental models, ECM1 expression correlates inversely with disease severity (97).

**Figure 4:**
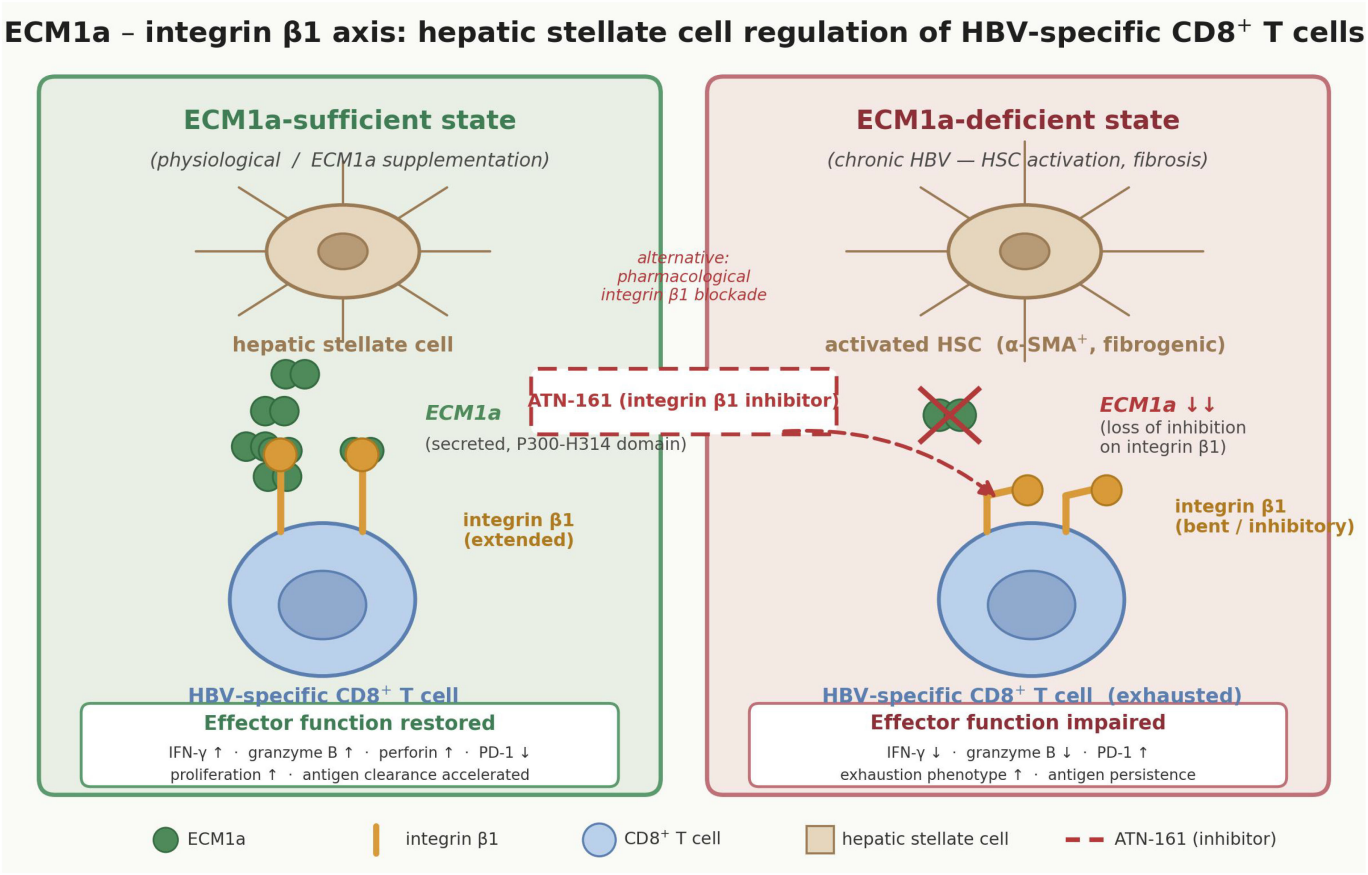
ECM1a–integrin β1 axis: hepatic stellate cell regulation of HBV-specific CD8^+^ T cells. Quiescent hepatic stellate cells (HSCs) secrete ECM1a (containing the P300-H314 functional domain), which engages integrin β1 in its extended (active) conformation on adjacent CD8^+^ T cells. ECM1a binding releases the autoinhibitory tone exerted by integrin β1 on TCR signalling, supporting effector function — IFN-γ, granzyme B and perforin expression are restored, PD-1 declines, and HBV antigen clearance is accelerated (left). In chronic HBV with HSC activation and fibrosis (right), ECM1a is markedly down-regulated; integrin β1 adopts the bent (autoinhibitory) conformation, exhaustion is reinforced and antigen persists. Pharmacological integrin β1 blockade with ATN-161 (Ac-PHSCN-NH_2_) provides an alternative route to relieving this inhibition and is currently under preclinical investigation as an adjunct to existing immunotherapies.

In CHB, hepatic ECM1 declines progressively with disease, leading to αv integrin-mediated activation of latent TGF-β by hepatocytes and hepatic stellate cells (HSCs) (97, 98). Once activated, TGF-β not only drives HSC activation and fibrosis but profoundly suppresses HBV-specific CD8+ T-cell function — reducing IFN-γ and granzyme B production, biasing differentiation towards Foxp3+ Tregs, and constraining effector-molecule expression (27, 45, 93). The ECM1-TGF-β-T-cell-exhaustion axis therefore constitutes a hepatic-specific immunoregulatory circuit, and restoration of ECM1 function or pharmacological TGF-β antagonism are emerging therapeutic strategies. Indeed, the 2024 study by Heim et al. explicitly identified TGF-β antagonism as a candidate strategy for restoring attenuated effector HBV-specific T cells (45) (**Figure 4**).

Integrin-targeted agents are at an earlier stage. ATN-161 (Ac-PHSCN-NH_2_), a non-RGD α5β1 antagonist peptide originally developed as an antiangiogenic agent (137, 138), inhibits tumour growth and angiogenesis in mouse models (137) and has completed phase I trials in solid tumours (138). Although direct evidence in HBV is limited, the central role of α5β1 and αv integrins in TGF-β activation and hepatic microenvironment biology (98, 139, 140) suggests that integrin antagonism — especially with hepatic-targeted delivery — could indirectly relieve T-cell suppression by reducing TGF-β activation. Reed et al. showed that αvβ1 inhibition substantially blunts hepatic fibrosis (139), and Henderson et al. demonstrated that αv integrins on hepatocytes and HSCs are central regulators of fibrogenesis (140), providing the mechanistic groundwork for testing integrin antagonists in HBV-specific immunology.

The “hepatic immune rheostat” described by Bosch et al. (2024) opens an entirely new dimension of HBV immunoregulation (46). They reported that dysfunctional CXCR6+ CD8+ T cells accumulating in chronic HBV exhibit elevated cAMP-response-element modulator (CREM) activity — a feature distinct from canonical exhaustion. Increased CREM activity rendered the cells unresponsive to TCR stimulation. LSECs were identified as the cellular driver of this CREM programme, constituting a “rheostat” for hepatic CD8+ T-cell activity (46). Targeting CREM or its upstream LSEC signals therefore represents a therapeutic avenue distinct from existing approaches.

Co-stimulatory receptors such as 4-1BB (CD137) and OX40 (CD134) provide a complementary perspective on HBV immunotherapy (44, 141). As outlined in Section 1, 4-1BB or OX40 agonism converts intrahepatically primed dysfunctional HBV-specific CD8+ T cells into bona fide antiviral effectors with markedly greater efficacy than inhibitory-receptor blockade (44); Jacobi et al. independently demonstrated synergy of OX40 agonism with PD-L1 blockade in HBeAg-negative CHB (141). Together these data herald a strategic shift from “release of suppression” to “active engagement” that may define the next generation of HBV immunotherapy.

The ECM1–latent-TGF-β axis, the hepatic immune rheostat (LSEC–CREM–CXCR6+ CD8+ T cells) and the 4-1BB/OX40 co-stimulatory circuit identify HBV-specific T-cell dysfunction as organ-tailored rather than a transplant of the LCMV exhaustion programme. These axes nominate uniquely hepatic therapeutic vulnerabilities — recombinant ECM1a, hepatocyte-targeted αv-integrin antagonism, CREM/LSEC-axis interference, and 4-1BB/OX40 agonism — that operate outside the conventional PD-1/PD-L1 paradigm and define a new tier of intervention targets specific to chronic HBV infection.

## 4 Strategies for reversing HBV-specific T-cell exhaustion

### 4.1 Immune checkpoint blockade (ICB)

**Figure 5:**
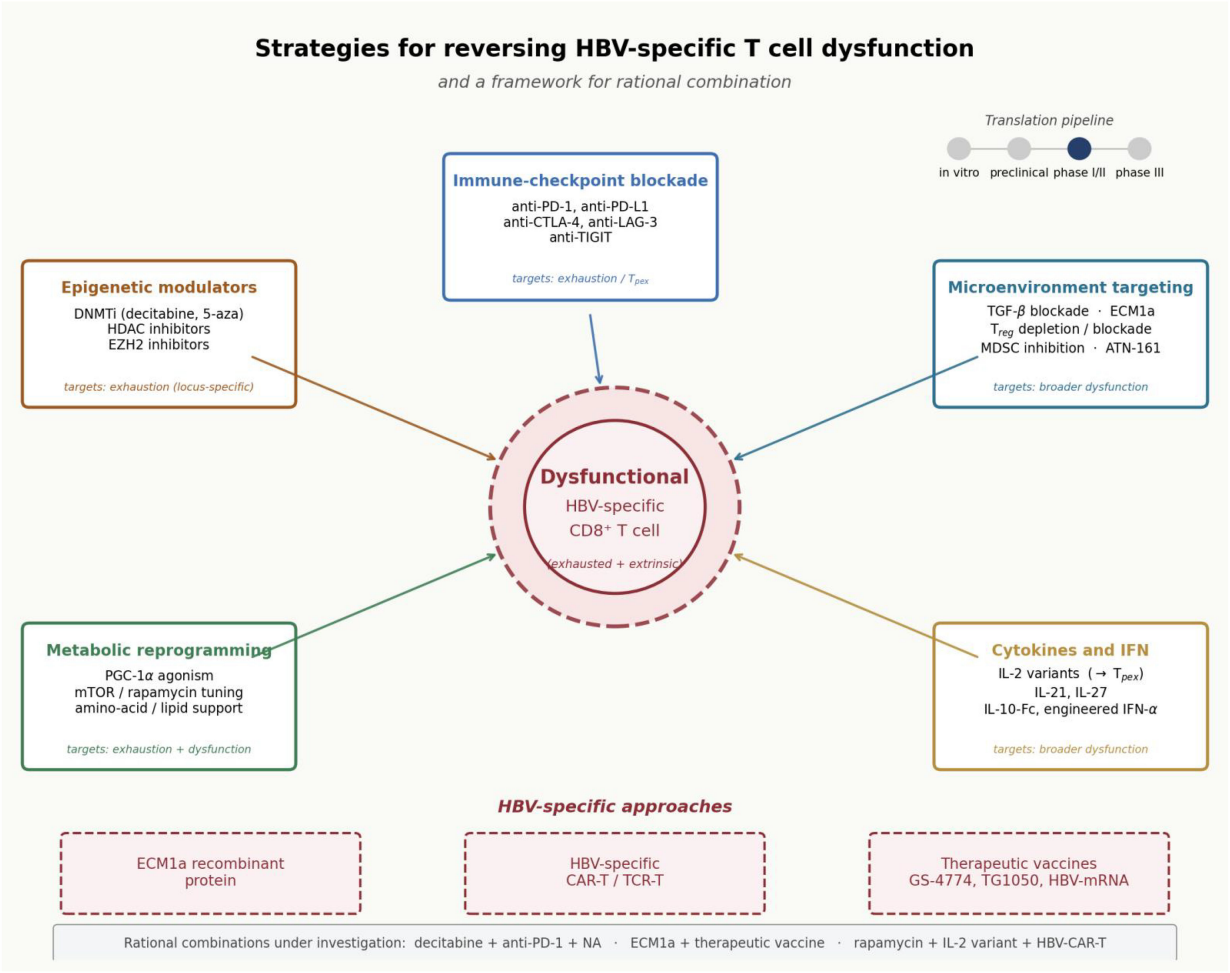
Strategies for reversing HBV-specific T cell dysfunction and a framework for rational combination. The dysfunctional HBV-specific CD8^+^ T cell is placed at the centre, reflecting the textual distinction in Sections 3–4 between TOX-mediated exhaustion and exhaustion-independent dysfunction. Five intervention classes converge on this dysfunctional cell: immune-checkpoint blockade (anti-PD-1, anti-PD-L1, anti-CTLA-4, anti-LAG-3, anti-TIGIT; flagged as primarily targeting the TOX-defined exhaustion arm and the T_pex reservoir), epigenetic modulators (DNMTi such as decitabine and 5-azacytidine, HDAC inhibitors, EZH2 inhibitors; locus-specific effects on the exhaustion programme), metabolic reprogramming (PGC-1α agonism, mTOR/rapamycin tuning, amino-acid and lipid support; relevant to both exhausted and more broadly dysfunctional T cells), cytokines and interferons (IL-2 variants targeting T_pex, IL-21, IL-27, IL-10–Fc and engineered IFN-α; mostly addressing broader dysfunction), and microenvironment-directed agents (TGF-β blockade, ECM1a, T_reg depletion or blockade, MDSC inhibition, integrin β1 blockade with ATN-161; addressing the extrinsic, exhaustion-independent component of dysfunction). HBV-specific approaches — recombinant ECM1a, HBV-targeted CAR-T or TCR-T cells and therapeutic vaccines (GS-4774, TG1050, HBV-mRNA) — provide additional levers and are aligned at the bottom of the figure. Most candidates remain in preclinical or early clinical development; rationally designed combinations (decitabine + anti-PD-1 + nucleos(t)ide analogue; ECM1a + therapeutic vaccine; rapamycin + IL-2 variant + HBV-CAR-T) currently represent the most promising translational paradigms.

**Figure 5** summarizes the principal classes of reversal strategy and a framework for their rational combination, and **Table 2** lists representative agents together with their molecular targets, stage of development and supporting evidence. ICB was rapidly translated from oncology into HBV research after its dramatic successes in cancer (38, 39, 54, 55, 142). PD-1/PD-L1 blockade is the most extensively studied modality. Early ex vivo studies showed that anti-PD-L1 antibodies partially restore IFN-γ production and proliferation in peripheral and intrahepatic HBV-specific CD8+ T cells from CHB patients (38, 39, 55). Bengsch et al. systematically compared responses across CHB phases and reported that inactive carriers — who retain a more functional T-cell pool — derive the greatest benefit, whereas patients with high viraemia and long-standing infection, in whom terminal exhaustion predominates, respond poorly (54).

**Table 2:** Representative strategies for reversing HBV-specific T-cell exhaustion.

Strategy class	Agent/approach	Target	Model/stage	T-cell effect	Limitations	Refs
Immune checkpoint blockade	Nivolumab (anti-PD-1)	PD-1	CHB phase I (NCT02942082)	Partial restoration of HBV-specific CD8+ function; HBsAg decline 5–15%	irAEs/hepatitis; limited monotherapy efficacy	(143)
	Envafolimab/ASC22 (anti-PD-L1)	PD-L1	CHB phase IIb	~6% HBsAg loss; up to 22% in low-baseline (<100 IU/mL) subgroup	Requires patient stratification	—
	Anti-TIGIT/LAG-3/Tim-3	TIGIT/LAG-3/Tim-3	Preclinical/early clinical	Synergistic with anti-PD-1	Limited human CHB data	(56–58, 145)
	RG6084 (small-molecule PD-L1 inhibitor)	PD-L1	CHB phase I	Hepatocyte-targeted T-cell reactivation	Early-trial hepatotoxicity signal	—
Epigenetic modulators	Decitabine (DAC)	DNMT1/3a	Murine HBV/CHB-PBMC ex vivo	Demethylation of effector loci; restored IFN-γ/granzyme B	Genome-wide effects; HCC risk in cirrhosis	(127)
	Vorinostat/panobinostat	HDAC	HIV/oncology; HBV preclinical	Augments reversal when combined with vaccine/ICB	Myelotoxicity; cccDNA effect unclear	(123)
	EZH2 inhibitors (e.g. tazemetostat)	PRC2/H3K27me3	Oncology; HBV preclinical	Reverses repressive marks at effector loci	Approved in oncology; preclinical in HBV	(123)
Engineered cytokines	IL-2v/pH-sensitive IL-2	IL-2Rβγ	Preclinical (LCMV/tumour)	Selective Tpex expansion without Treg expansion	Safety/dose optimisation in progress	(71, 72)
	IL-21, IL-27	IL-21R/IL-27R	CHB human and mouse data	Sustains Tpex, IFN-γ	Therapeutic dose not established in CHB	(148, 149)
	Hepatocyte-targeted IFN-α2 variants	IFNAR	Engineered preclinical	Hepatic specificity, reduced systemic toxicity	Manufacturing complexity	(147)
Microenvironment-directed	Anti-TGF-β (galunisertib, etc.)	TGF-β/TGFβR	Oncology; HBV preclinical	Relieves Tpex suppression; synergy with PD-1 blockade (Heim 2024)	Cardiovascular/fibrosis-related risk	(8, 97, 98, 139)
	ATN-161 (αv-integrin antagonist)	αv integrin	Phase I oncology	Disrupts integrin-mediated TGF-β activation	Limited HBV data	(137, 138)
	ECM1 mimetic/restoration	Latent TGF-β/αv integrin	Murine fibrosis models	Maintains TGF-β latency; antifibrotic	No clinical compound yet	(97)
	Treg/MDSC depletion (low-dose CY, anti-CD25)	Treg/MDSC	CHB and tumour models	Relieves local immunosuppression	Autoimmune risk; non-specific	(89–91, 94, 95)
Metabolic rewiring	PGC-1α/mitochondrial activators	Mitochondrial biogenesis	Tumour/chronic infection/HBV	Restores OXPHOS; NAC/MitoTEMPO restore IFN-γ	Lack of specific compounds	(68, 133, 134)
	Rapamycin/mTOR modulation	mTORC1	Adoptive T-cell preclinical	Promotes memory phenotype	Immunosuppression at high dose	(151)
	L-arginine/arginase inhibitors	Arginine metabolism	Preclinical	Restores T-cell metabolism; counters MDSC arginine depletion	Local vs systemic dosing unclear	(94, 132)
Adoptive cell therapy	HBV-specific TCR-T (mRNA)	S/Core/Pol epitopes	First-in-human HBV-related HCC	Direct elimination of HBV+ hepatocytes	Cytokine-release syndrome; off-target risk	(155, 160)
	HBV-specific CAR-T (HBsAg-targeted)	HBsAg	Murine HBV chimeric models	Antiviral activity, hepatocyte clearance	Hepatotoxicity; persistence concerns	(153, 154, 156, 157, 159)
Therapeutic vaccines	GS-4774 (yeast-based)	S/Core/X antigens	CHB phase II	Induces T-cell responses; greater effect with ICB	Monotherapy fails to lower HBsAg	(143, 161)
TLR agonists	Vesatolimod (TLR7)/selgantolimod (TLR8)	TLR7/TLR8	CHB phase II	Innate-immune activation; indirect T-cell support	Limited monotherapy HBsAg decline	(162–165)
Combination regimens	NA + DAC + anti-PD-1 (triplet)	Multi-axis	Proof of concept	Synergistic functional restoration	No randomised CHB data yet	(8, 127, 143)
	NA + siRNA + ICB sequential	Multi-axis	Phase II (REEF series)	Antigen reduction followed by immune activation	Optimal sequencing under study	(153, 155, 165, 166)
	4-1BB/OX40 agonism + ICB	4-1BB/OX40 + PD-1	Murine HBV (Andreata 2024)	Microenvironmental remodelling; T-cell reactivation	Not yet in clinic	(7, 141)

Reference numbers correspond to the bibliography. “—” indicates that no major paper for the agent (e.g. ASC22, RG6084) is currently included in the bibliography. Abbreviations: NA, nucleos(t)ide analogue; ICB, immune checkpoint blockade; DAC, decitabine; HDAC, histone deacetylase; DNMT, DNA methyltransferase; MDSC, myeloid-derived suppressor cell; Tpex, precursor exhausted T cell; Ttex, terminally exhausted T cell; OXPHOS, oxidative phosphorylation; irAE, immune-related adverse event.

Ferrando-Martinez et al. provided complementary cellular insight: PD-L1 blockade rescues HBV-specific responses chiefly in low-exhaustion populations (LAG3−TIM3−PD-1+), but is ineffective when terminal exhaustion is dominant (142). These findings highlight the importance of patient stratification and reinforce the rationale for combination therapy.

In the clinic, the phase I trial by Gane et al. of nivolumab combined with the GS-4774 therapeutic vaccine first established the safety and preliminary efficacy of ICB in CHB (143): among 12 NA-suppressed patients, one achieved sustained HBsAg loss and most exhibited HBsAg decline, without severe hepatotoxicity. Although immune-related hepatitis (irAH) is a theoretical concern, hepatic adverse events were manageable in this carefully selected, low-dose cohort.

Envafolimab (ASC22, KN035), a subcutaneously administered PD-L1 antibody developed in China, has shown distinctive promise in CHB. In the phase IIb trial of Qian et al. (NCT04465890), 1 mg/kg envafolimab every other week for 24 weeks in NA-suppressed patients with baseline HBsAg ≤10,000 IU/mL produced significantly greater HBsAg decline than placebo, with a notable HBsAg-loss rate among the subgroup with baseline HBsAg <100 IU/mL and an acceptable safety profile (144). RG6084 (RO7191863), a hepatocyte-targeted antisense oligonucleotide that degrades PDCD1LG1 (PD-L1) mRNA via RNase H, expanded hepatic CD8+ and NK-cell pools in preclinical models and entered phase I clinical evaluation in CHB (NCT04225715); a hepatic adverse-event signal led to dose modification in the early-phase study and the programme is still under clinical evaluation. Such liver-restricted strategies, in principle, deliver checkpoint relief locally without systemic immunotoxicity, and represent the precision arm of HBV immunotherapy.

Combinatorial blockade of multiple inhibitory receptors — PD-1+CTLA-4, PD-1+LAG-3, PD-1+TIGIT — is well validated in oncology (145). Schurich et al. first showed that CTLA-4 blockade, alone or with PD-1, restores apoptosis-prone CD8+ T-cell function in CHB (53). Liu et al. demonstrated synergy of PD-L1 blockade with therapeutic vaccination in animal models (146). Cai and colleagues confirmed that TIGIT blockade independently restores HBV-specific T-cell function and synergises with PD-1 blockade (58). Jacobi et al. showed that OX40 agonism combined with PD-L1 blockade augments HBV-specific CD4+ T cells in HBeAg-negative CHB (141).

Three core limitations of ICB remain. First, terminally exhausted cells (Ttex) respond poorly to single-agent ICB. Second, immune-related hepatitis can occur, possibly because checkpoint relief unleashes non-specific T-cell activation. Third, responses are typically partial and transient, reflecting the persistence of epigenetic locking (113, 124, 127). These limitations make combination strategies essential for functional cure.

### 4.2 Epigenetic modulators

Given the persistence of epigenetic scars after HCV cure (124–126), epigenetic modulators are now considered indispensable for genuine functional rescue. DNMT and HDAC inhibitors are the most extensively studied agents.

Decitabine (DAC), a low-toxicity, low-dose DNMT inhibitor, has shown promise in restoring exhausted T-cell function (127). Ghoneim et al. showed that DAC erases methylation marks at the PDCD1 locus and that, when combined with PD-L1 blockade, substantially augments IFN-γ and granzyme B production in chronic LCMV (127). In HBV, ex vivo PBMC studies indicate that DAC plus HBV-peptide stimulation enhances HBV-specific CD8+ T-cell responses, although clinical trial data in CHB are still lacking (27).

HDAC inhibitors (HDACi) have similar appeal (123). Henning and colleagues comprehensively summarised the role of HDACi in T-cell effector differentiation, showing that retention of H3K27ac at effector loci promotes IFN-γ and granzyme B transcription (123). Chidamide (tucidinostat), a selective HDACi developed in China and approved there for peripheral T-cell lymphoma, warrants exploration in HBV-specific T-cell remodelling.

EZH2 inhibitors represent a distinct epigenetic axis. As the catalytic subunit of PRC2, EZH2 deposits H3K27me3 at effector loci and silences them in exhausted cells (123). EZH2 inhibition partially restores T-cell function in tumour models (123), and HBV-directed studies are now warranted.

The concept of “epigenetic preconditioning” is being extended to HBV. The premise is straightforward: epigenetic modulators are administered before or alongside immune-engaging therapy in order to lift the locking constraint and enable productive activation (123, 127). This mirrors strategies in tumour immunotherapy. Successful translation will require careful dosing, scheduling and patient selection to avoid off-target effects on healthy T-cell pools.

DNMT and HDAC inhibitors are mechanistically rational and supported by LCMV proof-of-concept, but three caveats apply to CHB. First, genome-wide demethylation in cirrhotic or pre-cirrhotic livers carries an unquantified HCC-promoting risk, given that patients eligible for CHB immunotherapy frequently harbour pre-malignant changes. Second, the locus-specificity of currently available agents is poor; targeted epigenetic editing platforms (dCas9-TET1, dCas9-DNMT3A) remain preclinical, with no validated *in-vivo* delivery system for HBV-specific T cells. Third, no clinical trial in CHB has yet evaluated DAC dosing or scheduling relative to NA suppression and checkpoint blockade, leaving optimal combination architecture undefined.

### 4.3 Engineered interferons and cytokines

IFN-α remains the only conventional antiviral with demonstrable HBsAg-clearing potential, owing to its dual antiviral and immunomodulatory effects (9, 13). Conventional PegIFN-α achieves HBsAg loss in a fraction of patients when used in NA combination or sequential regimens, but response rates are modest and adverse events are common. Engineering of IFN-α has therefore become an active area of development, aimed at reducing systemic toxicity, increasing hepatic specificity, and enhancing T-cell rescue.

PD-L1 induction is one important caveat. Tan et al. reported that STAT1 activation during IFN-α therapy upregulates hepatic PD-L1, partially counteracting the immune-activating effects of IFN-α (147). Next-generation IFN-α2 variants therefore aim to retain antiviral and immune-stimulatory activity while attenuating PD-L1 induction and the drive towards T-cell exhaustion. Several engineered IFN-α2 variants are showing promise preclinically, including hepatocyte-targeted constructs (for example, NTCP-coupled formats) and receptor-affinity mutants engineered to dissociate antiviral activity from PD-L1 induction. Clinical validation in CHB is ongoing.

IL-10–Fc fusion proteins illustrate an instructive paradox. Although IL-10 is canonically immunosuppressive, Tang and colleagues showed that high-dose IL-10 paradoxically reactivates the metabolic and effector machinery of terminally exhausted CD8+ T cells. The development of IL-10–Fc has progressed substantially in oncology, but its application in HBV requires care given the dual role of IL-10 in CHB — it is secreted by Tregs, Bregs and Kupffer cells to maintain tolerance, yet may rejuvenate terminally exhausted T cells under specific conditions.

IL-2, IL-7 and IL-21 form a complementary trio of immunomodulatory cytokines (148, 149). IL-21, secreted by activated CD4+ T cells, sustains CD8+ T-cell proliferation, effector function and longevity. Publicover et al. showed that IL-21 governs the age-dependence of immune responses in murine HBV (148), and Ma et al. found that elevated serum IL-21 at week 12 of antiviral therapy correlated with HBeAg seroconversion (149). IL-2 variants engineered to bypass CD25 (IL-2v) selectively expand CD8+ T cells without expanding Tregs, and have outperformed wild-type IL-2 in chronic infection and tumour models. Their HBV translation is overdue.

IL-27 has emerged as a particularly attractive target. Venzin and colleagues showed in chronic HBV that intrahepatically primed HBV-specific CD8+ T cells require CD4+ T-cell help to “licence” Kupffer cells to secrete IL-27, which then restores effector function; exogenous IL-27 likewise rescues HBV-specific CD8+ T cells *in vivo* and ex vivo from CHB patients, establishing IL-27 as a potential druggable target (150).

Engineered cytokines (IL-2v, IL-10–Fc, IL-21, IL-27) and next-generation IFN-α variants offer biology-grounded mechanisms for direct T-cell rescue and are well aligned with the mechanistic targets identified in Section 3. The principal limitations are three-fold: (i) the narrow therapeutic window illustrated by the dual role of IL-10 (suppressive in CHB at endogenous doses but exhaustion-reversing at supraphysiological doses); (ii) immature hepatocyte- or T-cell-targeted delivery platforms, leaving systemic toxicity as a major concern; and (iii) the absence of validated PK/PD modelling for chronic-infection rather than oncology dosing, so optimal regimens for CHB remain to be empirically defined.

### 4.4 Microenvironment-directed strategies

The hepatic microenvironment harbours multiple suppressive elements that perpetuate HBV-specific T-cell exhaustion (22, 87, 88). Microenvironmental remodelling can therefore relieve T-cell suppression from outside the cell and create permissive conditions for cell-intrinsic interventions such as ICB and epigenetic therapy.

TGF-β antagonism is the most consequential of these strategies (45, 97, 98, 140). The 2024 study by Heim et al. firmly established TGF-β as the key restraint on attenuated effector HBV-specific CD8+ T cells (45). Available TGF-β-targeting tools include neutralising antibodies (for example, NIS793), TGFβR1 kinase inhibitors (for example, galunisertib), and αv-integrin antagonists, which act indirectly by blocking latent TGF-β activation (139, 140). Hepatocyte-targeted TGF-β antagonism — for example, GalNAc-conjugated siRNAs against TGFB1 — could deliver therapeutic activity with a more favourable safety profile.

Integrin-targeted agents offer a parallel route to microenvironmental remodelling. ATN-161 (Ac-PHSCN-NH_2_), a non-RGD α5β1 antagonist peptide, has completed phase I oncology trials with acceptable safety (137, 138). Given the central role of α5β1 and αv integrins in latent TGF-β activation and HSC biology (98, 139, 140), integrin antagonism may relieve T-cell suppression indirectly. Direct validation in HBV-specific immunology is needed, alongside hepatic-targeted delivery to limit systemic effects.

Treg modulation is an additional lever (89–91). Low-dose cyclophosphamide selectively impairs Treg expansion and has been used as an immunopotentiator in multiple settings. Chemokine-receptor antagonists (CCR4, CCR5) can block Treg recruitment to the liver. Systemic Treg depletion, however, risks autoimmunity, so localised, reversible Treg modulation is the preferred direction.

MDSC-directed strategies are also worth pursuing (94, 95). Pallett et al. demonstrated that G-MDSCs suppress HBV-specific T cells via Arg1-dependent arginine depletion (94); Arg1 inhibitors such as INCB001158 or arginine-axis modulation are therefore mechanistically rational. Alternatively, MDSCs can be differentiated into mature myeloid cells through agents such as all-trans retinoic acid (ATRA), an approach already exploited in oncology.

Microenvironmental remodelling addresses an upstream driver of exhaustion and may unlock the field where ICB monotherapy has under-performed. However, the agents in this class — TGF-β neutralising antibodies, αv-integrin antagonists, Arg1 inhibitors, low-dose cyclophosphamide and chemokine-receptor antagonists — carry substantial off-target liabilities (cardiovascular effects with pan-TGF-β blockade, broad immunological consequences of systemic T_reg or MDSC depletion, and the autoimmunity risk inherent in pan-T_reg modulation). Hepatic-restricted delivery (GalNAc-conjugated siRNAs, hepatocyte-targeted antibodies) is conceptually attractive but largely unvalidated. Direct clinical experience in CHB is essentially absent.

### 4.5 Metabolic rewiring strategies

Metabolic rewiring is a frontier reversal axis with strong synergy potential alongside epigenetic and checkpoint interventions (67, 68, 134). The principle is to restore mitochondrial function, redirect substrate utilisation, and relieve microenvironmental metabolic competition, thereby providing the energetic and biosynthetic foundation for T-cell recovery.

PGC-1α agonists are the central tool for restoring mitochondrial biogenesis (133). Scharping et al. first showed that the tumour microenvironment suppresses T-cell PGC-1α expression and that retroviral PGC-1α overexpression restores mitochondrial function and effector capacity in tumour-infiltrating lymphocytes (133). Comparable strategies in HBV are at an early stage but are expected to gain traction as a metabolic mainstay.

Rapamycin (mTOR inhibition) plays a context-dependent role in T-cell differentiation (151). At low doses, mTOR inhibition skews differentiation towards central memory, enhancing metabolic flexibility and longevity; Araki and colleagues showed that rapamycin enhances memory formation and vaccine responses in chronic LCMV (151). Excessive mTOR inhibition, in contrast, blunts T-cell activation. Precise dose calibration is therefore critical for HBV applications.

Glutamine-axis modulation is a newer strategy. Glutamine is a key amino-acid source for T cells, and its modulation can steer T cells towards specific lineages. Pre-infusion glutamine conditioning has shown promise in adoptive-cell therapy by enhancing antitumour efficacy (152). Whether comparable conditioning could improve HBV-specific CAR-T or TCR-T outcomes deserves investigation.

Arginine supplementation directly addresses the MDSC-Arg1 axis identified by Pallett et al. (94, 132). Geiger and colleagues showed that exogenous L-arginine improves T-cell metabolism and antitumour function in preclinical models (132). Whether nutritional intervention or Arg1 inhibitors can improve arginine availability and HBV-specific T-cell function in CHB warrants formal clinical evaluation.

Mitochondrial ROS scavengers carry direct immunological promise: Fisicaro et al. showed that MitoTEMPO and N-acetylcysteine (NAC) partially restore IFN-γ and granzyme B in HBV-specific CD8+ T cells (68). With NAC already in long-standing clinical use, this class is well placed for accelerated translational development (68).

Metabolic rewiring offers a foundational layer that should synergise with checkpoint and epigenetic interventions, and at least one agent (NAC) has decades of clinical use. The limitations are that mechanism-specific drugs (PGC-1α agonists, mitochondrial-dynamics modulators) remain preclinical, that rapamycin requires fine dose calibration to avoid net T-cell suppression, and that nutritional or amino-acid-pathway interventions (L-arginine, glutamine, IDO/TDO inhibitors) lack rigorous HBV-specific trial data. Whether metabolic intervention alone can deliver clinically meaningful HBsAg decline — rather than serving as adjuvant — is unlikely on current evidence.

### 4.6 Adoptive cell therapy and therapeutic vaccines

When the endogenous HBV-specific T-cell repertoire is profoundly exhausted or oligoclonal, adoptive transfer of exogenous T cells offers an alternative (153–158). The two principal approaches are HBV-specific CAR-T and TCR-T cells.

The CAR-T concept was first proposed for HBV by Bohne et al. (2008), who fused HBsAg-specific scFv to CD3ζ and co-stimulatory domains (CD28 or 4-1BB) and showed cytolytic activity *in vitro* and in HBV-transgenic mice (153). Krebs et al. developed second- and third-generation HBV-CAR-Ts and demonstrated potent viral control in AAV-HBV mice (154). Festag and colleagues built fully humanised HBV-CAR constructs and validated them in immunocompetent mouse models (159).

TCR-T leverages native TCR specificity to address a wider epitope repertoire (155, 156). Wisskirchen et al. used TCR grafting to introduce high-affinity HBV-specific TCRs into autologous lymphocytes, achieving substantial viral suppression and HBsAg reduction in HBV-infected humanised mice (160). Tan and colleagues used mRNA electroporation to express HBV-specific TCRs transiently, sidestepping the long-term safety concerns of permanent CAR-T integration, and reported initial clinical activity in HBV-related HCC (155).

Exhaustion remains the central obstacle for HBV-directed cell therapy: even highly functional infused cells may rapidly succumb to the high-antigen hepatic environment. Pre-engineering against exhaustion — through TOX deletion, PGC-1α overexpression, or mTOR modulation — is therefore likely to define next-generation HBV CAR-T/TCR-T products.

Therapeutic vaccines aim to “re-awaken” or rebuild HBV-specific immunity. Representative products include GS-4774 (yeast-based Tarmogen), TG1050 (MVA vector encoding multiple HBV antigens) and HepTcell (peptide vaccine) (161). The randomised phase II study by Lok et al. showed that GS-4774 monotherapy did not significantly reduce HBsAg but did induce HBV-specific T-cell responses in some patients (161). Combining GS-4774 with PD-1 blockade (nivolumab) yielded greater immune activation (143), suggesting synergy between therapeutic vaccines and ICB.

TLR agonists, including TLR7 (vesatolimod) and TLR8 (selgantolimod) ligands, represent a complementary class of innate-immune activators that indirectly enhance HBV-specific T-cell responses (162–165). Boni et al. were the first to show that TLR7 agonism augments HBV-specific T-cell and NK-cell responses in NA-treated CHB patients (162). Selgantolimod has now completed phase II evaluation with acceptable safety and preliminary signals of efficacy (165).

HBV-specific TCR-T and CAR-T strategies bypass the endogenous exhausted repertoire and have generated proof-of-concept antiviral activity in humanised models. However, two HBV-specific obstacles do not arise in oncology: (i) the hepatic high-antigen environment rapidly drives infused cells into the same exhaustion state they were designed to circumvent, so pre-engineering against exhaustion (TOX deletion, PGC-1α overexpression) is likely required; and (ii) HBsAg-targeted CAR-T can in principle produce diffuse hepatocyte damage if HBsAg expression is broadly distributed across the parenchyma. Therapeutic vaccines, in turn, deliver antigen to a host whose antigen-specific compartment is already deeply suppressed — explaining their consistent monotherapy failure and motivating combination regimens with checkpoint or innate-immune agonists. TLR7/8 agonists offer broad innate-immune activation but have shown only modest HBsAg decline as monotherapy and provoke transient flu-like symptoms.

## 5 Clinical translation: current status and challenges

### 5.1 ICB in CHB: from expectation to clinical reality

PD-1/PD-L1 blockade is the most successful immunotherapy paradigm in oncology, but its translation into CHB has been slower. Gane et al. reported the first phase I trial of single-dose nivolumab (0.3 mg/kg) in NA-suppressed, non-cirrhotic CHB patients in 2019: of 22 participants, only one achieved HBsAg loss, and most exhibited only modest HBsAg decline (143). Subsequent phase IIa studies confirmed that monotherapy is of limited efficacy, with only 5–15% of patients achieving ≥1 log10 IU/mL HBsAg decline by week 24 and no clear dose dependence (143).

Envafolimab (ASC22), a PD-L1 antibody developed in China, has shown more encouraging early-phase data. In the phase IIb trial reported by Qian et al. (NCT04465890), 60 NA-suppressed CHB patients with baseline HBsAg ≤10,000 IU/mL received subcutaneous envafolimab for 24 weeks; HBsAg loss was observed in 3 of 60 (approximately 5%) of treated patients, with the greatest decline concentrated in those with baseline HBsAg <100 IU/mL (in whom the HBsAg-loss rate reached approximately 22%) (144). This finding crystallises a critical principle: low-baseline HBsAg patients are most likely to benefit from ICB, suggesting that ICB should be deployed as consolidation rather than induction therapy. RG6084 (RO7191863), a hepatocyte-targeted antisense oligonucleotide that degrades PDCD1LG1 mRNA via RNase H, has been evaluated in a phase I CHB trial (NCT04225715); an early-trial hepatotoxicity signal led to dose modification, and the programme remains under clinical evaluation.

A central concern in CHB is the risk of immune-related adverse events (irAEs), particularly hepatotoxicity. PD-1 blockade in cancer is associated with immune-related hepatitis in 5–10% of patients, with grade 3–4 hepatic injury in 1–2% (143). Against the backdrop of an inherently inflamed liver, ICB-induced hepatotoxicity in CHB could be amplified. To date, published CHB-ICB trials have demonstrated acceptable overall safety, but sample sizes are limited and most trials have excluded patients with cirrhosis, viraemia or autoimmune comorbidity (143, 167). How to deploy ICB safely in broader CHB populations — especially those with cirrhosis or HBV DNA positivity — remains an unresolved question.

At a deeper level, the limited efficacy of single-agent ICB reflects the multilayered nature of HBV-specific T-cell exhaustion (113, 114, 124). Pauken et al. and Sen et al. showed that PD-1 blockade restores only a fraction of exhausted T-cell function, while epigenetic imprints persist (113, 114). Ghoneim et al. extended this work in murine models to demonstrate that PD-1 blockade alone cannot reverse *de novo* DNMT3A-driven methylation locking (116) — implying that combinatorial epigenetic intervention is required to genuinely unlock the exhaustion programme.

### 5.2 Epigenetic intervention combined with NAs: into the demethylation era

Low-dose decitabine (DAC) combined with NAs has shown promise in preclinical studies for augmenting HBV-specific T-cell responses (127). Mechanistically, DAC may not only reverse intrinsic DNA-methylation locking in T cells but also activate endogenous retroviral elements (ERVs), triggering “viral mimicry” and elevating intrahepatic type-I interferon signalling and T-cell infiltration.

Two core challenges constrain DAC translation. First, as a non-selective genome-wide demethylating agent, DAC may simultaneously reactivate tumour-suppressor and proto-oncogenic loci — a particular concern in CHB-cirrhosis populations with elevated baseline HCC risk. Second, the optimal DAC regimen — dose, frequency, and sequencing relative to NAs and ICB — has not been defined in CHB, hampering the design of phase II/III trials.

HDAC inhibitors such as vorinostat and panobinostat have a robust track record in HIV latency reversal and have been studied extensively as “shock and kill” agents. In HBV, analogous “cccDNA activation and elimination” strategies remain at proof-of-concept stage, but combinations of HDACi with HBV-specific adoptive T-cell transfer have demonstrated preliminary activity in mouse models (123).

### 5.3 Therapeutic vaccines and immune agonists: monotherapy and combination strategies

Therapeutic vaccine development now spans more than three decades, evolving from conventional protein vaccines to DNA, viral-vectored and mRNA platforms (143, 165). The phase II study of GS-4774, a heat-inactivated yeast vaccine expressing HBV S, X and core antigens, found that monotherapy failed to reduce HBsAg significantly but did elicit HBV-specific T-cell responses in a subset of patients (143, 165). TG1050, an adenovirus type-5 vector encoding HBV core, polymerase and HBs, was well tolerated and induced HBV-specific T cells in NA-suppressed CHB patients in a phase Ib trial, but produced only modest HBsAg decline (11).

The limited monotherapy efficacy of therapeutic vaccines reflects the deep, pre-existing exhaustion of HBV-specific T cells in CHB patients: antigen alone cannot reactivate cells locked in this dysfunctional state (143, 165). Combination with ICB or other immune agonists has therefore become a central theme. The phase II study of GS-4774 with tenofovir showed enhanced induction of HBV-specific T-cell responses, suggesting that the low-antigen environment created by NAs may be permissive for T-cell rejuvenation (165).

TLR7/8 agonists represent a complementary class of innate-immune activators (166, 168–170). The TLR7 agonist vesatolimod (GS-9620) showed acceptable safety in NA-suppressed CHB patients in phase II, but induced only modest HBsAg decline (169). Phase II data for selgantolimod (TLR8 agonist GS-9688) showed HBsAg loss in approximately 5% of patients, with greater benefit in patients with baseline HBsAg <1000 IU/mL (166) — again confirming low-baseline HBsAg as a clinically relevant predictor of immune-therapy response.

### 5.4 Rationale for combination therapy: the necessity of multi-axis intervention

A consensus view has emerged that single-agent interventions are inadequate to overcome the multilayered architecture of HBV-specific T-cell exhaustion (30, 31, 34). Heim et al. (2024) showed directly that the “attenuated effector” compartment is restrained by TGF-β signalling and that neither PD-1 blockade nor TGF-β inhibition alone can fully reverse this dysfunction; combination of the two yields clear synergy (45), providing a direct mechanistic case for multi-axis therapy in CHB.

An ideal CHB functional-cure regimen should simultaneously achieve four objectives: (i) viral-load control through NAs or siRNAs to reduce antigen-driven exhaustion signalling; (ii) epigenetic unlocking via low-dose DAC or HDACi to release silenced effector loci; (iii) checkpoint relief through PD-1/PD-L1 or TIGIT blockade to restore effector function; and (iv) microenvironmental remodelling via TGF-β or integrin antagonism to attenuate local suppression (30, 31, 45, 91, 97).

A widely discussed “triplet” regimen consists of NAs (or siRNA) + epigenetic modulator + ICB (45, 127, 143). The logic is straightforward: NAs reduce antigen pressure, epigenetic modulators unlock the exhausted T cell, and ICB releases effector function. In murine models of persistent HBV, similar triplets have shown synergistic HBsAg clearance (127). However, no randomised CHB trial of such triplets has yet been reported, and both efficacy and safety remain to be established.

A second strategy of interest is sequential therapy: prolonged NA + siRNA suppression of viral antigens to extremely low levels, followed by short-term immune activation (ICB, therapeutic vaccine or CAR-T) to re-engage the T-cell compartment (153, 155, 165). The theoretical advantage is to avoid hepatotoxicity from immune activation in a high-HBsAg setting and to mimic the immunological trajectory of spontaneous HBsAg clearance. The REEF-1 and REEF-2 trials are currently exploring NA + siRNA + immune-agonist sequences (166) and will provide pivotal evidence on optimal combinations.

### 5.5 Translational obstacles: heterogeneity, biomarkers and dose optimisation

Patient heterogeneity is a fundamental obstacle to clinical translation (39, 41, 47, 52). Single-cell analyses have established that HBV-specific T cells display markedly distinct exhaustion phenotypes across the immune-tolerant, immune-active, inactive carrier and HBeAg-negative phases (52, 78). Heim et al. demonstrated that TOX+TCF1− terminally exhausted cells are enriched in HBeAg-negative hepatitis but relatively scarce in the immune-tolerant phase (79). This heterogeneity implies that uniform treatment approaches are likely to yield divergent outcomes across subgroups.

A second obstacle is the absence of validated biomarkers. Although PD-1^+^CD38^+^HLA-DR^+^ subsets and CD127↓ have been proposed as clinical markers of exhaustion (52, 80), no consensus, routine-laboratory-deployable biomarker yet predicts response to immunotherapy. The very low frequency of HBV-specific T cells in peripheral blood (typically <0.1%) further limits the utility of conventional flow cytometry or ELISpot assays in routine practice (8, 19) (**Table 3**).

**Table 3:** Immunological biomarkers of HBV-specific T-cell exhaustion and their potential clinical applications.

Category	Marker	Expression in CHB exhausted T cells	Clinical/prognostic significance	Detection method	Refs
Inhibitory receptors	PD-1 (PDCD1)	↑↑ Elevated on HBV-specific CD8+, particularly intrahepatic	Most-studied marker; also a bona fide activation marker, upregulated on effector CD8+ T cells in acute and early HBV infection; in chronic infection its sustained high-level expression in combination with TOX, TIM-3 and loss of TCF1 defines exhaustion; predicts anti-PD-1 response; correlates with HBeAg status	Flow (multimer + anti-PD-1)	(6, 54, 55, 59)
	CTLA-4	↑ Elevated on HBV-specific CD8+	Apoptosis-prone phenotype; anti-CTLA-4 target	Flow	(53)
	TIM-3	↑ Elevated; co-expressed with PD-1 on Ttex	Marker of deep exhaustion; combination ICB target	Flow	(56)
	LAG-3	↑ Elevated on intrahepatic HBV-specific T cells	Anti-LAG-3 target	Flow	(57, 145)
	TIGIT	↑ Elevated on HBV-specific CD8+	Predicts persistent HBV; combination ICB target	Flow	(58, 145)
	CD244 (2B4)	↑ Elevated on HBV-specific CD8+	Marks exhaustion; correlates with viral load	Flow	(6)
Transcription factors	TOX	↑↑ Elevated; defines exhaustion depth	Strongest single locking marker; correlates with CHB phase	Intracellular flow/IHC/scRNA-seq	(46, 47, 79, 99–101)
	TCF1 (TCF7)	↓ Reduced in Ttex; preserved in Tpex	Identifies Tpex (ICB-responsive subset)	Flow/scRNA-seq	(71, 75, 77, 78)
	T-bet (TBX21)	↓ Inverse to PD-1	Loss correlates with chronicity	Intracellular flow	(102)
	EOMES	↑ Paradoxically elevated in terminal Ttex	Marker of incompetent terminal state	Intracellular flow	(103)
	BLIMP-1 (PRDM1)	↑ Elevated in exhausted T cells	Drives exhaustion programme	Intracellular flow/scRNA-seq	(110)
	NR4A1/2/3	↑ Elevated	Reinforces exhaustion; potential editing target	scRNA-seq/qPCR	(104–106)
Surface/functional	CD127 (IL-7Rα)	↓ Reduced	Marks impaired memory transition	Flow	(80)
	CD38 + HLA-DR	↑ Double-positive HBV-specific CD8+	Activated–exhausted phenotype; predicts spontaneous control	Flow	(80)
	Granzyme B/perforin	↓ Reduced	Functional deficit	Intracellular flow/ICS	(33, 61)
	IFN-γ/TNF-α/IL-2 polyfunctionality	↓↓ Markedly reduced	Functional readout; predicts spontaneous control	ICS/ELISpot	(33, 61)
Metabolism	Mitochondrial mass/membrane potential (TMRM)	↓ Reduced	Bioenergetic insufficiency; metabolic intervention target	Flow (MitoTracker/TMRM)	(67, 68)
	Mitochondrial ROS	↑ Elevated	Reflects dysfunction; antioxidant-targetable	Flow (MitoSOX)	(68)
	Glucose uptake (2-NBDG)	↓ Reduced	Glycolytic insufficiency	Flow/Seahorse	(67, 128)
Epigenetics	PD-1 promoter methylation	↓ Hypomethylated	Imprinted exhaustion; persists after viral control	Bisulfite sequencing/methylation array	(116–118)
	IFNG/GZMB H3K27me3	↑ Elevated	Repressive chromatin mark of exhaustion	ChIP-seq/CUT&RUN	(114, 115, 120)
	Exhaustion-specific accessible chromatin	Altered ATAC profile	State transition; potential single-cell marker	scATAC-seq	(113, 114)
HBV-specific	Hepatic ECM1 expression	↓ Reduced in chronic HBV liver	Reflects loss of latent TGF-β regulation; correlates with fibrosis	IHC/serum ECM1 (under development)	(97)
	Plasma TGF-β1	↑ Elevated	Reflects TGF-β activity; correlates with fibrosis and exhaustion	ELISA	(8, 97, 98)
	qHBsAg level	↑↑ Elevated	Strongest predictor of response (low baseline ↔ better response)	Quantitative ELISA/chemiluminescence	(19, 165)
T-cell subsets	PD-1+CD38+HLA-DR+	↑↑ Elevated frequency	Activated-exhausted subset; potential stratification marker	Multi-colour flow	(80)
	Tpex (TCF1+PD-1+Slamf6+)	Variable frequency	Preferential ICB responder; expandable pool	Multi-colour flow/scRNA-seq	(71, 75, 78)
	Ttex (TCF1−TOX+Tim-3+)	↑ Frequency in late CHB	Resistant to standard ICB; combination target	Multi-colour flow/scRNA-seq	(46, 79)

Reference numbers correspond to the bibliography. Abbreviations: ICS, intracellular cytokine staining; ELISpot, enzyme-linked immunospot; ChIP-seq, chromatin immunoprecipitation sequencing; ATAC-seq, assay for transposase-accessible chromatin sequencing; scRNA/scATAC-seq, single-cell RNA/ATAC sequencing; TMRM, tetramethylrhodamine methyl ester; qHBsAg, quantitative HBsAg; Tpex, precursor exhausted T cell; Ttex, terminally exhausted T cell.

Liver biopsy can capture intrahepatic immune information but is too invasive for routine clinical decision-making (8). Future tools, modelled on circulating tumour-DNA technology, may include cell-free hepatic DNA (cf-hepDNA) methylation profiling, peripheral HBV-specific T-cell MHC-multimer staining, and spatial-transcriptomic-guided immune landscape mapping of the liver (123, 126).

Dose and schedule optimisation are equally critical. The Gane et al. trial used a nivolumab dose (0.3 mg/kg) far below standard oncological dosing, primarily to mitigate hepatotoxicity (143), but this may have under-stimulated the immune response. Striking the balance between sufficient immune activation and acceptable immunopathology will require additional dose-finding studies and PD/PK modelling. The optimal number of doses, the choice between continuous and intermittent dosing, and the sequencing of agents in combination regimens are also poorly defined.

Translating this mechanistic complexity into a clinical decision framework requires explicit consideration of the patient variables most likely to modify benefit and risk. We therefore provide a provisional mapping of intervention class to candidate patient subgroup, intended as a starting point for prospective testing rather than as a treatment guideline. Immune-checkpoint blockade is most plausibly beneficial in NA-suppressed, HBeAg-negative patients with low baseline qHBsAg (typically <100–1,000 IU/mL) and without active cirrhosis or autoimmune comorbidity, where the residual immune compartment retains TCF1+ progenitor exhausted cells and the hepatic-injury risk is lowest. Therapeutic vaccines (GS-4774, TG1050, mRNA constructs) are best suited to NA-suppressed patients in whom HBsAg has been pharmacologically reduced (siRNA/ASO platforms) so that newly induced T cells encounter a permissive antigen environment; HBeAg-positive, high-viraemia patients without preceding antigen reduction are unlikely to respond. Antigen-lowering platforms (bepirovirsen, JNJ-3989, NAPs) are most informative in patients with high baseline HBsAg, where the absolute opportunity for HBsAg decline is greatest, and are typically delivered on a background of NA suppression. Engineered cytokines targeting the Tpex pool (IL-2 variants, IL-21) and the LSEC–CREM/TGF-β axis (TGF-β inhibitors, integrin antagonists) are conceptually best matched to immune-active and HBeAg-negative phases where dysfunctional T cells co-exist with a still partially responsive Tpex reservoir. Adoptive transfer (TCR-T or CAR-T) is currently most defensible in HBV-related HCC or in CHB with cirrhosis when endogenous repertoires are exhausted or oligoclonal, but the high-antigen hepatic environment and on-target hepatocyte injury risk dictate cautious patient selection. Across all classes, additional modifiers — age, HBV genotype, genotype-specific HCC risk, fibrosis stage and the depth of NA-induced suppression — should be considered explicitly when designing trials. This framework is provisional and will require validation against multi-omic biomarkers as these mature; the goal is to make the patient–intervention pairings explicit so that the field can test them, rather than to recommend any specific therapy outside of a clinical trial.

## 6 Future directions and outlook

### 6.1 Single-cell and spatial multi-omics: precision stratification

Single-cell RNA sequencing (scRNA-seq), single-cell ATAC sequencing (scATAC-seq) and spatial transcriptomics are reshaping the map of HBV-specific T-cell exhaustion (39, 41, 47, 78, 79). These technologies resolve heterogeneity at subset resolution and capture spatial interactions between T cells and parenchymal cells, LSECs, Kupffer cells and HSCs (97). Heim et al. (2024) used spatial transcriptomics to delineate the spatial distribution of attenuated effector T cells and their TGF-β-dependent context, providing precision targets for microenvironmental intervention (45).

Within the next 5–10 years, single-cell- and spatial-omics-based immune classification is likely to supersede traditional clinical-phase staging as the primary framework for guiding CHB immunotherapy. Stratifying patients by intrahepatic Tpex/Ttex ratios, TOX intensity and depth of epigenetic locking will probably distinguish “readily reversible”, “deeply locked” and intermediate immune phenotypes, each of which will require a tailored intervention (78, 79).

### 6.2 Beyond unlocking: epigenetic erasure and lineage reprogramming

Current epigenetic modulators such as DAC and HDACi are essentially “unlocking” agents — they release genes silenced by methylation or histone marks (117–119). However, HCV-cure studies establish that profoundly exhausted T cells retain epigenetic scars even after antigen elimination, suggesting that unlocking alone may be insufficient for full functional restoration (124–127).

A more ambitious strategy is “epigenetic erasure” — targeted removal of methylation marks at specific loci using precision tools such as dCas9-TET1 or dCas9-DNMT3A fusions (122). In principle, this approach can rewrite the T-cell epigenome and restore the developmental potential of memory-like cells. Two challenges loom large: *in vivo* delivery to HBV-specific T cells, and avoidance of off-target effects on genomic stability. A complementary concept is lineage reprogramming — engineering the transcription-factor circuit (for example, TCF1 or TBX21 overexpression or modulation) to redirect exhausted cells towards a memory-like or Tpex state (71, 72, 75). Wieland and colleagues showed that TCF1+ memory-like CD8+ T cells in chronic HCV preserve functional capacity (77), providing initial feasibility for TCF1-centred reprogramming in HBV.

A more forward-looking direction is the use of induced pluripotent stem cell (iPSC) technology to generate HBV-specific T cells (13, 48). Transducing HBV-specific TCRs into iPSCs followed by T-lineage differentiation could, in principle, produce unlimited supplies of “youthful”, exhaustion-naïve HBV-specific T cells for adoptive transfer (155, 158), although the strategy remains conceptual.

### 6.3 Microenvironmental engineering: from passive accommodation to active redesign

The hepatic microenvironment’s tolerogenic disposition is a major reason for HBV persistence (62, 87, 88). The classical assumption is that T cells must adapt to the liver, but recent work indicates that active remodelling of the microenvironment can break tolerance at its source (45, 46). The “hepatic immune rheostat” described by Bosch et al. (2024) reveals that intrahepatic CD8+ T cells engage CREM-mediated local immunoregulation that limits excessive immunopathology (46). Targeting CREM or upstream LSEC signalling could therefore offer a means to lift hepatic tolerance.

ECM1, secreted by hepatocytes, suppresses intrahepatic immune activation by maintaining TGF-β in its latent form (97). Fan et al. demonstrated that ECM1 is downregulated in CHB liver, leading to aberrant TGF-β activation, T-cell suppression and fibrosis (97). ECM1 mimetics or TGF-β inhibitors therefore present clear therapeutic opportunities. Better delineation of hepatocyte–immune-cell–HSC interactions will reveal further microenvironmental targets.

### 6.4 Redefining functional cure: from HBsAg loss to immune-homeostatic restoration

The clinical definition of CHB functional cure currently centres on sustained HBsAg loss (6, 7, 9, 10) — a tractable, quantifiable endpoint that may, however, fall short of capturing immunological cure. Some patients with HBsAg loss still harbour low-level cccDNA and residual T-cell abnormalities (7), indicating that serological cure is not necessarily immunological cure.

In light of the deeper mechanistic understanding now available, we propose that CHB functional cure should be defined across three dimensions: (i) virological cure — sustained HBsAg loss, undetectable HBV DNA, and minimal or undetectable intrahepatic cccDNA; (ii) immunological cure — restoration of HBV-specific T- and B-cell responses to levels approximating those of natural resolvers, with marked downregulation of TOX and substantial unlocking of epigenetic constraints; and (iii) clinical cure — reversal or stabilisation of fibrosis, with HCC risk declining towards that of the general population (6, 10, 126).

This expanded definition has direct implications for trial design. Future CHB immunotherapy trials should incorporate not only HBsAg loss as a primary endpoint, but also multi-omic readouts — cccDNA quantification, multiparameter T-cell function assays, and depth of epigenetic locking — as secondary endpoints (7, 126). Standardising and validating these endpoints will be a defining task for the field.

### 6.5 Cross-disease lessons: HCV cure, HIV cure and tumour immunology

The successful cure of HCV through DAA therapy provides a powerful reference for CHB (124–127). The studies by Yates, Hensel and Tonnerre showed that even after HCV elimination, HCV-specific T cells retain durable epigenetic scars and persistent functional deficits (124–127). The central lesson is unambiguous: viral antigen clearance alone does not restore T-cell function, and active epigenetic intervention may be indispensable.

HIV cure research, with its “shock and kill” and “block and lock” paradigms, provides additional conceptual scaffolding for HBV cure. HDAC inhibitors, PD-1 blockade and broadly neutralising antibodies have all advanced into clinical testing in HIV. Tumour immunotherapy, with TILs, CAR-T, TCR-T and bispecific T-cell engagers (BiTEs) approved or in late-stage trials (153, 155, 161, 162), has accumulated practical experience that translates well to CHB despite the biological differences between persistent infection and cancer. Both share a common backbone — T-cell exhaustion (30, 31, 34) — and many oncological lessons (irAE management, predictive biomarkers, combination optimisation) can be imported wholesale.

## 7 Conclusion

HBV-specific T-cell exhaustion is a multilayered and epigenetically locked state, orchestrated by transcription factors (TOX, NFAT, NR4A), enforced by DNA methylation and histone modifications, sustained by mitochondrial metabolic insufficiency, and reinforced by liver−specific microenvironmental cues including the ECM1/TGF−β axis and the hepatic immune rheostat. Single−axis interventions such as PD−1 blockade alone are therefore mechanistically insufficient. Achieving functional cure requires rational, multi−axis combinations: antigen load reduction (NAs, siRNA), epigenetic unlocking (DAC, HDACi), checkpoint relief (anti−PD−1/PD−L1, anti−TIGIT), microenvironmental remodelling (TGF−β inhibition, ECM1 mimetics), engineered cytokines (IL−2v, IL−10–Fc), and, in selected cases, CAR−T or TCR−T therapy. Clinical translation now depends on (i) multi−omic precision stratification to distinguish “readily reversible” from “deeply locked” exhaustion; (ii) mechanism−driven regimens, notably the NA/siRNA + epigenetic modulator + ICB triplet; (iii) refined management of immune−related adverse events and hepatotoxicity; and (iv) an expanded definition of functional cure that incorporates immunological and epigenetic benchmarks alongside virological endpoints.

## Glossary

AASLD: American Association for the Study of Liver Diseases
ASO: antisense oligonucleotide
ATAC-seq: assay for transposase-accessible chromatin sequencing
ATRA: all-trans retinoic acid
BiTE: bispecific T-cell engager
BLIMP1: B-lymphocyte-induced maturation protein 1
CAM: capsid assembly modulator
CAR-T: chimeric antigen receptor T cell
cccDNA: covalently closed circular DNA
CHB: chronic hepatitis B
CREM: cAMP response element modulator
CTLA-4: cytotoxic T-lymphocyte-associated protein 4
DAA: direct-acting antiviral
DAC: decitabine
DNMT: DNA methyltransferase
DNMTi: DNMT inhibitor
EASL: European Association for the Study of the Liver
ECM1: extracellular matrix protein 1
EOMES: eomesodermin
EpiCyTOF: epigenome-guided mass cytometry
ERV: endogenous retrovirus
EZH2: enhancer of zeste homologue 2
G-MDSC: granulocytic MDSC
HBV: hepatitis B virus
HCC: hepatocellular carcinoma
HDAC: histone deacetylase
HDACi: HDAC inhibitor
HSC: hepatic stellate cell
ICB: immune checkpoint blockade
ICOS: inducible T-cell co-stimulator
ICS: intracellular cytokine staining
IDO: indoleamine 2,3-dioxygenase
IFN: interferon
iPSC: induced pluripotent stem cell
irAE: immune-related adverse event
irAH: immune-related hepatitis
LAG-3: lymphocyte-activation gene 3
LCMV: lymphocytic choriomeningitis virus
LSEC: liver sinusoidal endothelial cell
LTC: latent TGF-β
MDSC: myeloid-derived suppressor cell
mTOR: mechanistic target of rapamycin
NA: nucleos(t)ide analogue
NAC: N-acetylcysteine
NAP: nucleic acid polymer
NFAT: nuclear factor of activated T cells
NK: natural killer
NR4A: nuclear receptor subfamily 4 group A
OXPHOS: oxidative phosphorylation
PD-1: programmed cell death-1
PD-L1: programmed death-ligand 1
PegIFN-α: pegylated interferon-α
PGC-1α: peroxisome proliferator-activated receptor γ
PRC2: polycomb repressive complex 2
ROS: reactive oxygen species
scATAC-seq: single-cell ATAC sequencing
scRNA-seq: single-cell RNA sequencing
siRNA: small interfering RNA
SVP: subviral particle
T-bet: T-box transcription factor TBX21
TCF1: T-cell factor 1
TCR: T-cell receptor
TCR-T: engineered TCR T cell
TDO: tryptophan 2,3-dioxygenase
TGF-β: transforming growth factor β
TIGIT: T-cell immunoreceptor with Ig and ITIM domains
TIM-3: T-cell immunoglobulin and mucin-domain containing-3
TLR: Toll-like receptor
TNF-α: tumour necrosis factor α
TOX: thymocyte selection-associated high mobility group box
T_pex: precursor exhausted T cell
T_reg: regulatory T cell
TRM: tissue-resident memory T cell
TSL: stem-like dysfunctional T cell
T_tex: terminally exhausted T cell
WHO: World Health Organization
